# Comparative efficacy and safety of odanacatib, abaloparatide, denosumab, teriparatide, and bisphosphonates for male osteoporosis: a systematic review and network meta-analysis

**DOI:** 10.3389/fendo.2026.1836818

**Published:** 2026-05-08

**Authors:** Chengwu Lu, Libao Zhang, Changhui Xue, Mingxin Liao, Jichang Fei, Xiaojun Chen, Linfeng Wang

**Affiliations:** 1 The Affiliated Nanping First Hospital of Fujian Medical University, Nanping, China; 2 Fujian Minbei Orthopedic Research Institute, Nanping, China; 3 The 907th Hospital of Chinese PLA Joint Logistics Support Force, Nanping, China

**Keywords:** abaloparatide, adverse events, bisphosphonates, bone mineral density, denosumab, male osteoporosis, network meta-analysis, teriparatide

## Abstract

**Background:**

Evidence from randomized controlled trials (RCTs) on pharmacological management of primary osteoporosis in men is fragmented, making direct comparisons across drug classes difficult, and head-to-head evidence remains limited. We performed a network meta-analysis (NMA) to compare the relative efficacy and safety of commonly used anti-osteoporotic agents in men.

**Methods:**

We systematically searched PubMed, Web of Science, and the Cochrane Library from inception to December 2025 for RCTs enrolling men with primary osteoporosis. Eligible interventions included oral bisphosphonates (OBP), intravenous bisphosphonates (IBP), abaloparatide (ABA), denosumab (DEN), teriparatide (TER), and odanacatib (ODN), compared with placebo/usual care (PLA/CTRL) or active treatments. Primary efficacy outcomes were changes in bone mineral density (BMD) at the lumbar spine (LS), total hip (TH), and femoral neck (FN), preferentially extracted at the time point closest to 12 months (range: 6–24 months). Safety outcomes were any adverse events (AEs) and serious adverse events (SAEs). Pairwise meta-analyses were conducted in Stata 18.0, and NMA in R 4.3.1 under a consistency framework. Treatments were ranked using SUCRA. The protocol was registered in PROSPERO (CRD420261279572).

**Results:**

Twenty-one RCTs (n = 4,409) were included. Compared with PLA/CTRL, TER and ABA significantly improved FN-BMD (TER: MD 2.97, 95% CI 0.74–5.20; ABA: MD 2.83, 95% CI 0.11–5.55), and OBP also showed benefit (MD 1.61, 95% CI 0.15–3.07). For TH-BMD, significant gains were observed for OBP (MD 2.25, 95% CI 1.70–2.81), ABA (MD 2.13, 95% CI 1.43–2.83), and TER (MD 1.12, 95% CI 0.35–1.89); OBP showed a greater increase than TER (MD 1.13, 95% CI 0.32–1.95). For LS-BMD, ABA (MD 7.31, 95% CI 4.25–10.37), TER (MD 6.97, 95% CI 4.53–9.42), and OBP (MD 3.43, 95% CI 1.84–5.02) were superior to PLA/CTRL, and both ABA and TER outperformed OBP (ABA vs OBP: MD 3.88, 95% CI 0.44–7.33; TER vs OBP: MD 3.55, 95% CI 1.08–6.01). Most head-to-head comparisons across active agents were inconclusive with wide uncertainty. Node-splitting suggested no significant local inconsistency (P > 0.05). Safety comparisons for AEs and SAEs were largely imprecise; SUCRA rankings should therefore be interpreted cautiously.

**Conclusions:**

In men with primary osteoporosis, treatment effects on BMD differ by skeletal site: ABA and TER yield larger gains at the lumbar spine, whereas OBP demonstrates robust improvements at the total hip; evidence for femoral neck improvement is clearer for TER and ABA versus PLA/CTRL. However, the extent to which these BMD improvements translate into fracture risk reduction remains uncertain because direct fracture endpoint data were limited in the included RCTs. Safety evidence also remains limited due to imprecision, and SUCRA rankings should therefore be interpreted alongside effect estimates and their uncertainty. Further high-quality, long-term RCTs focusing on fracture outcomes and drug-specific adverse events are warranted.

## 1 Introduction

Osteoporosis is a systemic metabolic bone disease characterized by reduced bone mass, microarchitectural deterioration, and decreased bone strength, with fragility fracture as its most serious clinical consequence (1–3). Compared with women, osteoporosis in men is more likely to be underrecognized and undertreated in routine clinical practice. However, once fragility fractures occur, men face substantial risks of disability, subsequent fractures, and mortality, leading to a considerable individual and societal burden (4–6). Notably, osteoporotic fractures are common among middle-aged and older adults, and many patients are not identified or diagnosed until after a fracture event, underscoring the need to establish a clearer evidence base on the benefits and risks of pharmacotherapy in men (7–10).

Current pharmacological treatments for osteoporosis can be broadly categorized by mechanism into two main strategies: antiresorptive therapies and anabolic/osteoanabolic therapies (11–15). The interventions included and compared in the present study comprised the following classes. First, selective cathepsin K inhibitors, represented by odanacatib (ODN), reduce bone resorption by inhibiting a key osteoclast protease, thereby improving bone mass and strength (13, 14). Second, the monoclonal antibody denosumab (DEN) exerts potent antiresorptive effects primarily by inhibiting the RANKL signaling pathway, leading to reduced osteoclast formation and activity (12). Third, recombinant hormone/osteoanabolic agents, including teriparatide (TER) and abaloparatide (ABA), promote bone formation and improve bone microarchitecture through pathways involving the parathyroid hormone 1 receptor (PTH1R) (12, 16). Fourth, bisphosphonates, including oral bisphosphonates (OBP) and intravenous bisphosphonates (IBP), inhibit osteoclast-mediated bone resorption and are widely used as foundational therapies with substantial accumulated evidence (14, 15).

Although odanacatib (ODN) was once considered a promising antiresorptive agent, its clinical development was ultimately discontinued because of safety concerns (14, 16). This background underscores the importance of evaluating safety outcomes alongside efficacy when comparing pharmacological treatments for male osteoporosis.

Nevertheless, randomized controlled trial (RCT) evidence in male osteoporosis remains relatively fragmented, and direct head-to-head comparisons across drugs are limited, making it difficult to draw reliable conclusions regarding comparative effectiveness based on individual trials alone. Therefore, we conducted a network meta-analysis (NMA) to systematically compare the relative efficacy and safety of OBP, IBP, ABA, DEN, TER, and ODN in men with primary osteoporosis, and to rank competing interventions using the surface under the cumulative ranking curve (SUCRA), thereby providing a more comprehensive evidence base to inform individualized treatment decisions.

## 2 Materials and methods

### 2.1 Search strategy

This study is a systematic review and network meta-analysis (NMA). The conduct and reporting followed the PRISMA statement and its extension for network meta-analysis (PRISMA-NMA) and adhered to methodological recommendations from the Cochrane Handbook to enhance transparency and reproducibility (17). The protocol was prospectively registered in PROSPERO (registration number: CRD420261279572), with the research question, eligibility criteria, outcomes, and statistical plan predefined before study initiation.

We systematically searched PubMed, Web of Science, and the Cochrane Library from inception to December 2025. The search strategy comprised three components: (1) population/disease terms (e.g., male/men, primary osteoporosis), (2) intervention terms (e.g., abaloparatide, denosumab, teriparatide, odanacatib, bisphosphonates and synonyms), and (3) study design terms (e.g., randomized controlled trial), combining controlled vocabulary and free-text terms. The full search strings, fields, and Boolean logic are provided in the **Supplementary Materials**. To minimize the risk of missing relevant studies, no formal search of gray literature sources, clinical trial registries, or unpublished data repositories was performed. To reduce the risk of missing relevant studies, we also conducted backward citation tracking of relevant systematic reviews and meta-analyses and manually screened potentially eligible records.

### 2.2 Inclusion criteria and study design

Two investigators (CWL, LBZ) independently screened studies in two stages (title/abstract screening followed by full-text assessment) according to prespecified inclusion and exclusion criteria. For duplicate publications or multiple reports from the same cohort, we preferentially included the report with more complete information, longer follow-up, or more comprehensive outcome reporting. If different publications reported different outcomes from the same cohort, data were integrated while avoiding double counting of participants. Disagreements were resolved by discussion with a third investigator (LFW).

Only randomized controlled trials (RCTs) were included. Case reports, reviews, conference abstracts, letters, commentaries, protocols, and other non-original studies were excluded. Participants were restricted to men with primary osteoporosis; studies focusing on secondary osteoporosis due to hypogonadism, glucocorticoid use, malignancy, or other systemic diseases were excluded. Eligible interventions included OBP, IBP, ABA, DEN, TER, and ODN. Comparators could be placebo/usual care controls (PLA/CTRL) or other active treatments. Trials involving combination therapy were excluded from quantitative synthesis if the independent effect of the target intervention could not be separated.

Efficacy outcomes were changes in bone mineral density (BMD) at the lumbar spine, total hip, and femoral neck, with preference given to percentage change in BMD. When absolute changes or different units were reported, outcomes were harmonized using consistent effect measures to ensure comparability (18–23). Safety outcomes included all adverse events (AEs) and serious adverse events (SAEs) (24, 25). AEs were defined as any unfavorable medical occurrence during the trial period, whereas SAEs were events meeting seriousness criteria (e.g., death, life-threatening events, disability, hospitalization, or prolonged hospitalization).

### 2.3 Data extraction and quality assessment

Two investigators independently extracted data and cross-checked for accuracy. Extracted items included first author, publication year, country/region, study design, sample size, follow-up duration, intervention details (dose, frequency, and route), comparator type, and numeric outcome data for efficacy and safety. When multiple follow-up time points were reported for the same outcome, we preferentially extracted data from the time point closest to 12 months to improve comparability across studies. If such a time point was not available, the prespecified primary endpoint or final follow-up was used. For studies with missing or incompletely reported variance data, we followed the recommendations in Section 6.5.2 of the Cochrane Handbook wherever possible to derive standard errors or standard deviations from available summary statistics (26). When studies reported between-group mean differences and P values, the corresponding standard errors were calculated using Cochrane-recommended formulas. When no measure of variance could be derived from the reported data, a standard deviation of 30 was imputed, following a pragmatic approach used in a published systematic review and meta-analysis. This approach was considered conservative in the sense that assigning a relatively large SD reduces the statistical weight of the affected study and helps avoid spuriously narrow confidence intervals arising from incompletely reported variance data (27). Sensitivity analyses were conducted where necessary to evaluate the robustness of primary findings to imputation assumptions.

Risk of bias for included RCTs was assessed using the Cochrane-recommended tool (28–30), covering random sequence generation, allocation concealment, blinding of participants/personnel, blinding of outcome assessment, incomplete outcome data, selective reporting, and other bias. Each domain was rated as low, high, or unclear risk. Assessments were performed independently by two investigators, with disagreements resolved by a third reviewer.

### 2.4 Statistical analysis

For outcomes with direct comparisons, we first conducted pairwise meta-analyses using Stata 18.0. Continuous outcomes were expressed as mean differences (MDs), and dichotomous outcomes as risk ratios (RRs), both with 95% confidence intervals (95% CIs). Between-study heterogeneity was quantified using I², and random-effects models were preferred when substantial heterogeneity was suggested; potential sources of heterogeneity were explored when feasible.

The NMA was performed in R 4.3.1 using the gemtc and BUGSnet packages (31–33) under a consistency framework, synthesizing direct and indirect evidence to estimate relative treatment effects (MDs for continuous outcomes; RRs for binary outcomes) with corresponding uncertainty intervals. Prior to NMA, the plausibility of the transitivity assumption was evaluated clinically and methodologically by examining baseline characteristics, follow-up duration, comparator settings, and measurement methods. Statistical inconsistency was assessed using global tests and local approaches (e.g., node-splitting). If inconsistency was detected, findings were interpreted in light of network structure and clinical differences, and sensitivity analyses were undertaken when necessary.

To describe relative treatment rankings for each outcome, we calculated SUCRA values and presented cumulative ranking probabilities and rank plots. If the number of included studies met methodological requirements, comparison-adjusted funnel plots were generated to explore publication bias or small-study effects; otherwise, potential bias was discussed descriptively. Results were presented using forest plots, league tables, network graphs, risk-of-bias plots, and SUCRA ranking plots. Risk-of-bias figures were optionally generated using Review Manager (RevMan 5.4) to improve readability and consistency, whereas primary computations were conducted in Stata 18.0 and R 4.3.1.

## 3 Results

**Figure 1:**
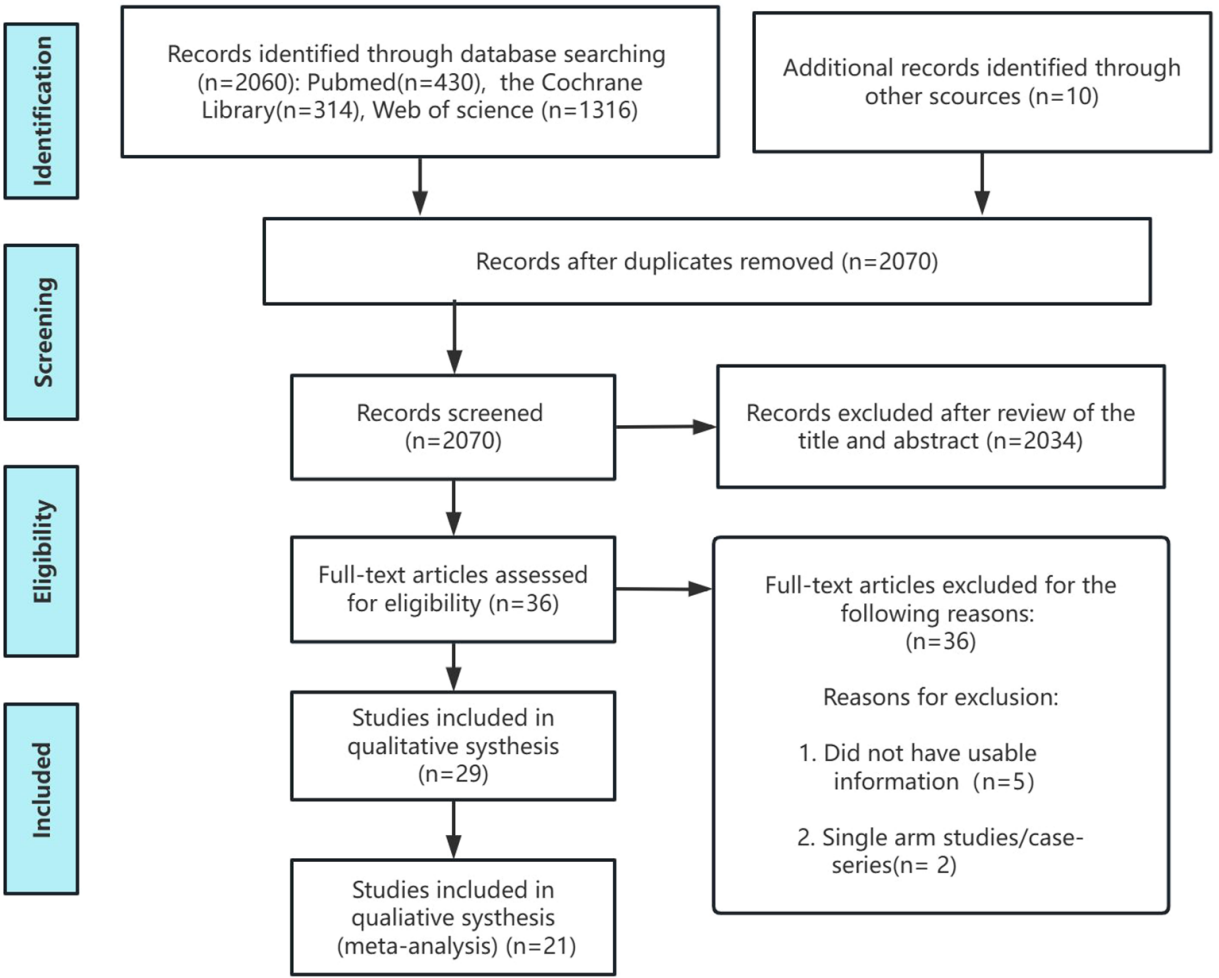
Flow chart of the selection process for relative studies in meta-analysis.

The study selection process is shown in **Figure 1**. The initial search identified 2,070 records; after removing duplicates, 36 records remained for title/abstract screening. Full texts of potentially eligible studies were assessed, and studies not meeting prespecified criteria were excluded (e.g., non-male primary osteoporosis populations, non-RCT designs, ineligible interventions, or missing key outcomes). Ultimately, 21 RCTs including 4,409 participants were included in the quantitative synthesis. Interventions encompassed OBP, IBP, ABA, DEN, TER, and ODN, with comparators including placebo and, in some trials, alfacalcidol, providing the evidence base for subsequent NMA.

**Figure 2:**
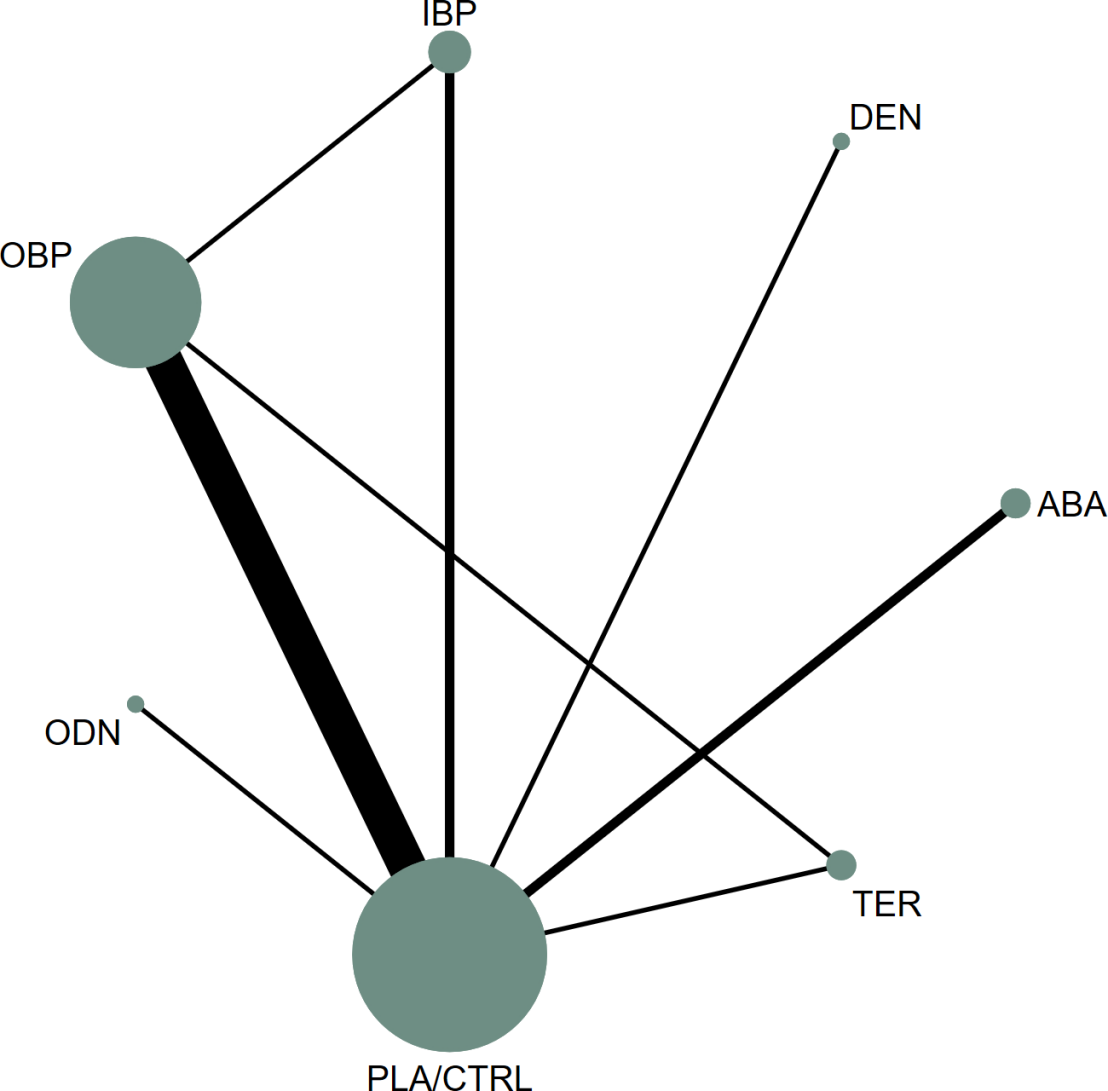
The network plot of all trials (ABA, abaloparatide; DEN, denosumab; TER, teriparatide; OBP, oral bisphosphonates; IBP, intravenous bisphosphonates; ODN, odanacatib; PLA/CTRL, placebo/control).

The network geometry is presented in **Figure 2**. Overall, each intervention was directly compared with placebo in at least one trial, and a subset of head-to-head trials among active agents enabled indirect comparisons across multiple treatments. Local inconsistency assessed by node-splitting did not indicate significant inconsistency (P > 0.05). As further shown in **Supplementary Table 1**, effect estimates were broadly similar between consistency and inconsistency models, suggesting adequate overall model fit and coherence of the evidence network.

Follow-up for the primary efficacy outcomes (lumbar spine, femoral neck, and total hip BMD) was predominantly 12 months, with a minority of studies reporting shorter or longer durations. To enhance comparability, we preferentially extracted outcomes closest to 12 months. Forest plots and network plots are provided in **Supplementary Figures 1**, **2**. In the network plots, node size reflects sample size and edge thickness reflects the number of studies contributing to each direct comparison. Visual inspection of funnel plots (**Supplementary Figure 3**) did not suggest marked asymmetry, indicating a potentially low risk of publication bias.

### 3.1 Study characteristics and study quality

Baseline characteristics of included studies are summarized in **Table 1** (34–54). Publication years ranged from 2000 to 2025. Sample sizes per trial ranged from 19 to 1,199 men, with follow-up durations mostly between 1 and 3 years. Most trials compared active agents with placebo and/or calcium plus vitamin D supplementation; some additionally used alfacalcidol as a comparator or co-intervention. Direct comparisons among active therapies were relatively limited, and network connectivity was largely established through comparisons against placebo/standard supplementation. Study settings were mainly from the United States, Germany, and China, in addition to some multicenter trials.

**Table 1:** The Main features of the articles.

Author, year	Study design	Country	Treatment	Comparator	Background treatment	Group/Patient	Length of interv
Binkley 2021	RCT	Fifty-six centers in 13 countries	Odanacatib 50 mg once weekly	Placebo	vitamin D3	G1: 128 G2: 115	104 weeks (2 years)
Nakamura 2014	RCT	Japan	Odanacatib 50 mg once weekly	Placebo	Ca and Vit D supplement	G1: 59 G2: 73	52 weeks
Ringe 2009	RCT	Germany	Risedronate 5 mg, oral daily administration	Daily alfacalcidol (1 microg)	Ca (1,000 mg) daily and Vit D (800 IU), daily oral administration	G1: 158 G2: 158	104 weeks (2 years)
Walker 2013	RCT	Columbia	Risedronate oral 35 mg, weekly oral administration	1: Teriparatide daily subcutaneous injection 20 µg 2: Combination of both	Ca (500 mg) and vit D (400 IU), daily oral administration	G1: 10 G2: 9 G3: 10	78 weeks (18 months)
Boonen 2009	RCT	Multicenter study	Risedronate 35 mg, weekly oral administration	Placebo	Ca (1 g) and vit D (400–500 IU), twice daily	G1: 191 G2: 93	104 weeks (2 years)
Orwoll 2010	RCT	USA	Ibandronate 150 mg, oral monthly administration	Placebo	Ca (1 g) and vit D (400 IU) twice daily	G1: 85 G2: 47	52 weeks (1 year)
Orwoll 2003	RCT	37 centers in 11 countries	Teriparatide 20ug, subcutaneous daily injection	1: Teriparatide 40ug, subcutaneous daily injection 2: Placebo	Ca (1000 mg) and Vit D (400–1200 IU), daily oral administration	G1: 151 G2: 139 G3: 147	52 weeks (1 year)
Qi 2021	RCT	China	Teriparatide 20 µg/day, daily subcutaneous injection	Alendronate 10 mg/day, oral daily administration	Ca and Vit D (dose not provided), daily oral administration	G1: 50 G2: 50	52 weeks (1 year)
Kaufman 2004	RCT	37 study sites in 11 countries	Teriparatide 20ug, subcutaneous daily injection	1: Teriparatide 40ug, subcutaneous daily injection 2: Placebo	Supplemental calcium (1,000 mg daily) and vitamin D (400–1,200 IU daily)	G1: 22 G2: 20 G3: 37	18 months
Boonen 2011	RCT	International	Zoledronic Acid 5 mg, yearly intravenous injection	Placebo	Ca (1–1.5 g) and Vit D (400–800 IU), daily administration	G1:248 G2: 260	104 weeks (2 years)
Boonen 2012	RCT	Multicenter study	Zoledronic acid 5 mg, yearly intravenous injection	Placebo	Ca (1 g) and Vit D (800–1000 IU), daily oral administration	G1: 588 G2: 611	104 weeks (2 years)
Orwoll 2010	RCT	North America, Australia	Zoledronic acid 5 mg, yearly intravenous injection	Alendronate 70 mg, oral daily administration	Ca (1 g) and Vit D (800–1000 IU), daily oral administration	G1: 154 G2: 148	104 weeks (2 years)
Czerwinski 2022	RCT	USA	Abaloparatide 80 µg, daily subcutaneous injection	Placebo	NR	G1: 149 G2: 79	52 weeks (1 year)
Matsumoto 2022	RCT	Japan	Abaloparatide 80 µg, daily subcutaneous self-injections	Placebo	Ca and Vit D supplement, daily oral administration	G1: 14 G2: 6	78 weeks (18 months)
Richard Eastell 2025	RCT	United Kingdom	Abaloparatide 80 µg, daily subcutaneous injection	Placebo	Ca and Vit D supplement, daily oral administration	G1: 114 G2: 64	52 weeks (1 year)
Orwoll 2012	RCT	Multicentre study (North America and Europe)	Denosumab 60 mg, sub cutaneous injection every 6 months (q6m)	Placebo	Ca (≥ 1 g) and Vit D (≥ 800 IU), daily oral administration	G1: 121 G2: 121	52 weeks (1 year)
Gonnelli 2003	RCT	Italy	Alendronate 10 mg, oral daily administration	No placebo	Ca (1000 mg) daily oral administration	G1: 39 G2: 38	156 weeks (3-years)
Hwang 2010	RCT	China	Alendronate 70 mg, oral weekly administration	No placebo	Ca and Vit D supplement, daily oral administration	G1: 23 G2: 23	24 weeks
Miller 2004	RCT	USA, Multicentre	Alendronate 70 mg, weekly oral administration	Placebo	Ca as carbonate (500 mg) and Vit D (200 IU), daily oral administration	G1: 109 G2: 58	52 weeks (1 year)
Orwoll 2000	RCT	20 centers in the United States and 10 other countries	Alendronate 10 mg, daily oral administration	Placebo	Ca (500 mg) and Vit D (400 IU), daily oral administration	G1:146 G2: 95	104 weeks (2 years)
Ringe 2004	RCT	Germany	Alendronate 10 mg, oral daily administration	Alfacalcidol (1 μg daily)	Supplemental calcium (500 mg daily)	G1: 68 G2: 66	3 years

Dosing regimens were generally consistent with routine clinical use: risedronate 35 mg weekly or 5 mg daily; alendronate 70 mg weekly or 10 mg daily; teriparatide 20 μg subcutaneously daily; denosumab 60 mg subcutaneously every 6 months; and odanacatib 50 mg once weekly. These standardized regimens provided a clinically comparable basis for assessing efficacy and safety across interventions.

**Figure 3:**
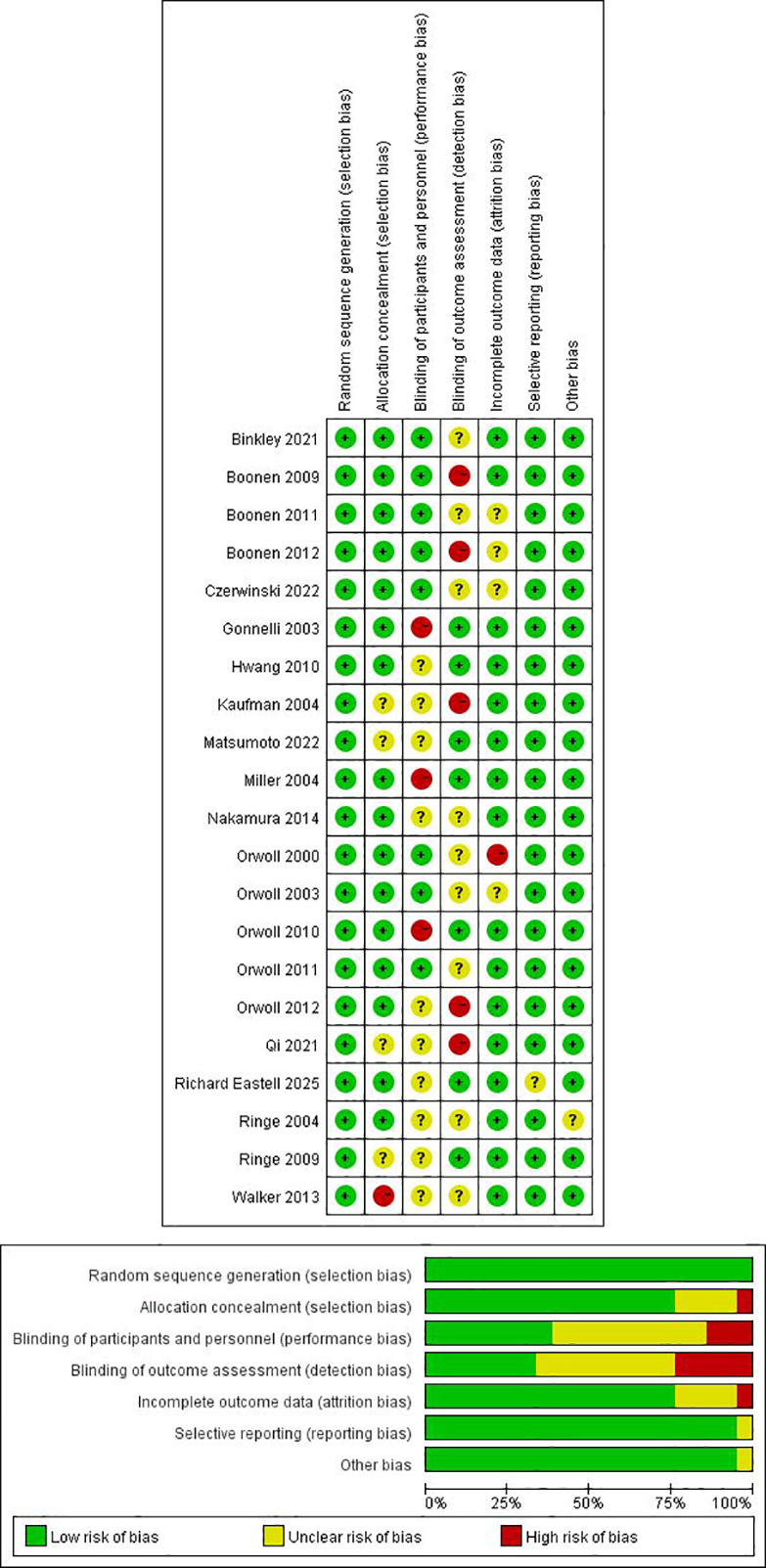
The network plot of all trials. Risk of bias summary for RCTs: Reviewers' judgments about each risk of bias item per included study.

Risk-of-bias assessments are shown in **Figure 3** based on the Cochrane approach. Overall, many trials provided insufficient details regarding random sequence generation and allocation concealment, particularly with respect to implementation and concealment procedures. In addition, selective outcome reporting was difficult to judge in some trials due to limited information on whether all prespecified outcomes were fully reported. Given these reporting limitations, many studies were rated as unclear risk in domains related to selection bias and reporting bias.

### 3.2 Primary outcomes

#### 3.2.1 Femoral neck BMD

**Figure 4:**
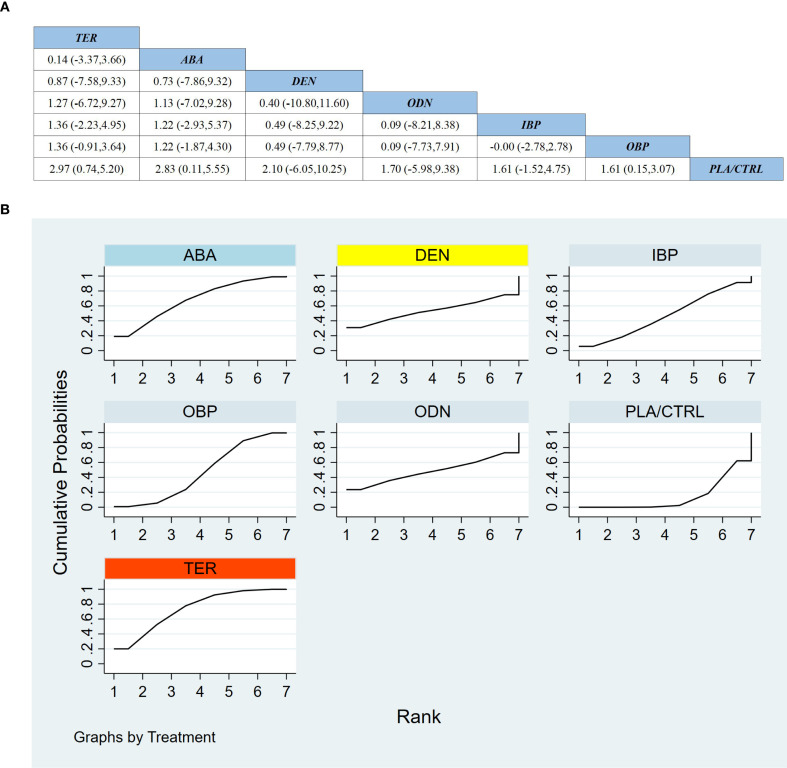
**(a)** The results of League table for Femoral neck BMD. **(b)** Ranking the probability of Femoral neck BMD percentage change.

Across 13 RCTs including 2,503 participants, we compared the effects of different treatments on FN-BMD. As shown in **Figure 4a**, compared with placebo/control (PLA/CTRL), TER achieved the largest statistically significant improvement in FN-BMD (MD 2.97, 95% CI 0.74 to 5.20). ABA also significantly increased FN-BMD (MD 2.83, 95% CI 0.11 to 5.55). Among bisphosphonates, OBP demonstrated a significant benefit versus PLA/CTRL (MD 1.61, 95% CI 0.15 to 3.07). In contrast, DEN, ODN, and IBP showed effect estimates favoring benefit but with wide CIs crossing the null (DEN: MD 2.10, 95% CI −6.05 to 10.25; ODN: MD 1.70, 95% CI −5.98 to 9.38; IBP: MD 1.61, 95% CI −1.52 to 4.75).

In the combined direct/indirect comparisons among active agents, most pairwise differences were not statistically significant (with most 95% CIs crossing 0). For example, TER vs ABA showed no clear difference (MD 0.14, 95% CI −3.37 to 3.66), and IBP vs OBP similarly showed no difference (MD −0.00, 95% CI −2.78 to 2.78), indicating remaining uncertainty regarding relative superiority among active treatments for FN-BMD.

Treatment ranking based on SUCRA is shown in **Figure 4b**: TER ranked first, ABA second, and DEN third. Notably, despite DEN ranking among the top three, its effect estimate versus PLA/CTRL had wide uncertainty and crossed the null, suggesting that rankings may be influenced by limited sample size and the number of available studies.

#### 3.2.2 Total hip BMD

**Figure 5:**
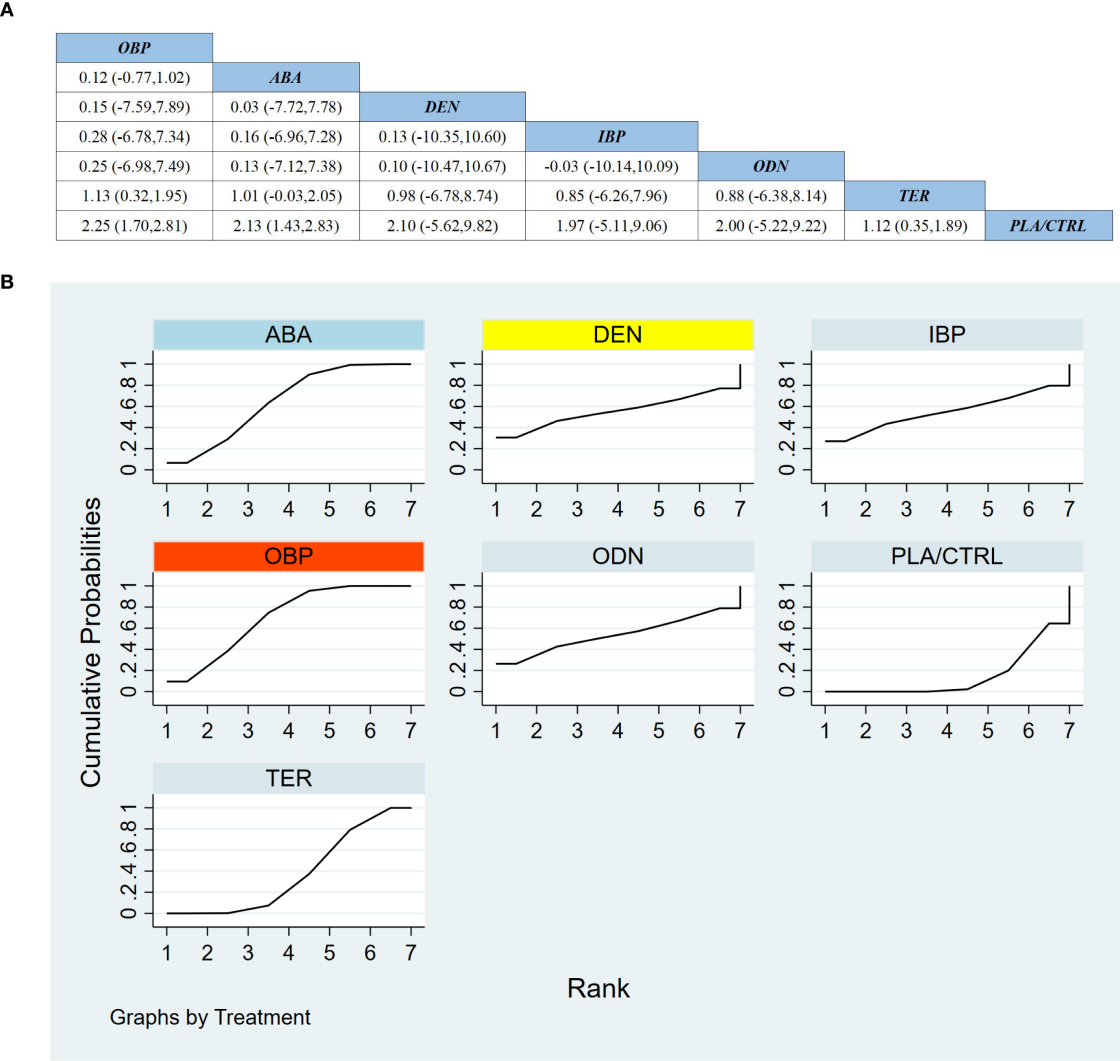
**(a)** The results of League table for Total hip BMD. **(b)** Ranking the probability of Total hip BMD percentage change.

Across 12 RCTs including 2,219 participants, we evaluated treatment effects on TH-BMD. As shown in **Figure 5a**, compared with PLA/CTRL, OBP, ABA, and TER significantly increased TH-BMD (OBP: MD 2.25, 95% CI 1.70 to 2.81; ABA: MD 2.13, 95% CI 1.43 to 2.83; TER: MD 1.12, 95% CI 0.35 to 1.89). In contrast, DEN, IBP, and ODN showed effect estimates favoring benefit but with wide CIs crossing 0 (DEN: MD 2.10, 95% CI −5.62 to 9.82; IBP: MD 1.97, 95% CI −5.11 to 9.06; ODN: MD 2.00, 95% CI −5.22 to 9.22).

Most pairwise comparisons between active agents were inconclusive (most 95% CIs crossed 0). Notably, OBP showed a greater gain in TH-BMD than TER (MD 1.13, 95% CI 0.32 to 1.95), whereas OBP vs ABA was not statistically significant (MD 0.12, 95% CI −0.77 to 1.02).

SUCRA rankings for TH-BMD (**Figure 5b**) indicated OBP as the highest-ranked treatment, followed by ABA and DEN. However, DEN’s estimate versus PLA/CTRL remained imprecise and crossed the null, suggesting potential uncertainty in its ranking due to limited evidence.

#### 3.2.3 Lumbar spine BMD

**Figure 6:**
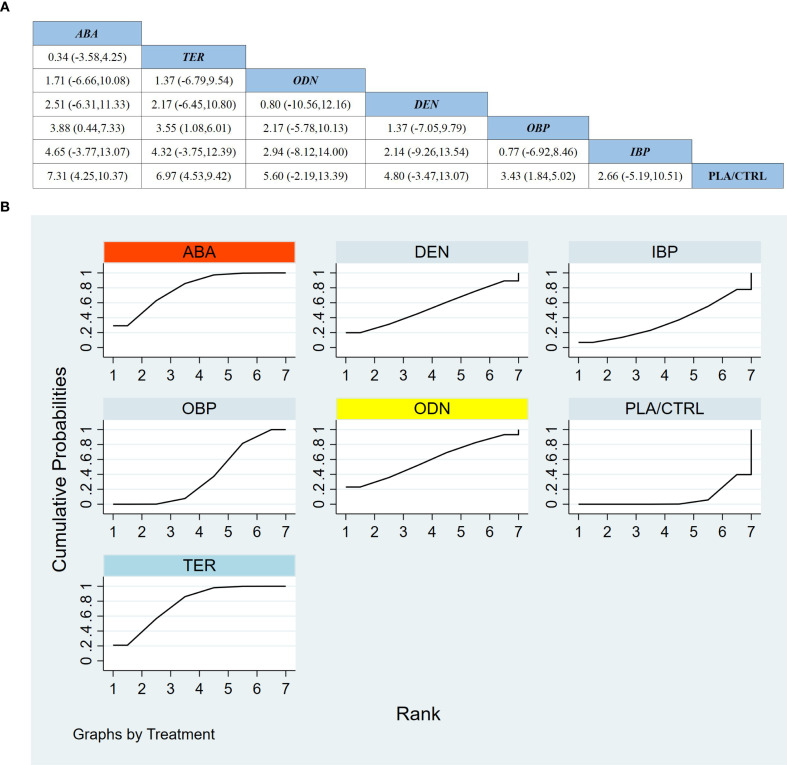
**(a)** The results of League table for Lumbar spine BMD. **(b)** Ranking the probability of Lumbar spine BMD percentage change.

Across 13 RCTs including 2,494 participants, we assessed treatment effects on LS-BMD. As shown in **Figure 6a**, compared with PLA/CTRL, ABA, TER, and OBP significantly improved LS-BMD (ABA: MD 7.31, 95% CI 4.25 to 10.37; TER: MD 6.97, 95% CI 4.53 to 9.42; OBP: MD 3.43, 95% CI 1.84 to 5.02). ODN, DEN, and IBP suggested potential benefit but with substantial uncertainty and non-significant estimates (ODN: MD 5.60, 95% CI −2.19 to 13.39; DEN: MD 4.80, 95% CI −3.47 to 13.07; IBP: MD 2.66, 95% CI −5.19 to 10.51).

In comparisons among active agents, both ABA and TER were superior to OBP (ABA vs OBP: MD 3.88, 95% CI 0.44 to 7.33; TER vs OBP: MD 3.55, 95% CI 1.08 to 6.01), whereas ABA vs TER showed no clear difference (MD 0.34, 95% CI −3.58 to 4.25). Most other comparisons were similarly inconclusive.

SUCRA rankings for LS-BMD (**Figure 6b**) placed ABA first, TER second, and ODN third; however, ODN-related estimates were imprecise and crossed the null, indicating persistent uncertainty.

### 3.3 Secondary outcomes

#### 3.3.1 All adverse events

**Figure 7:**
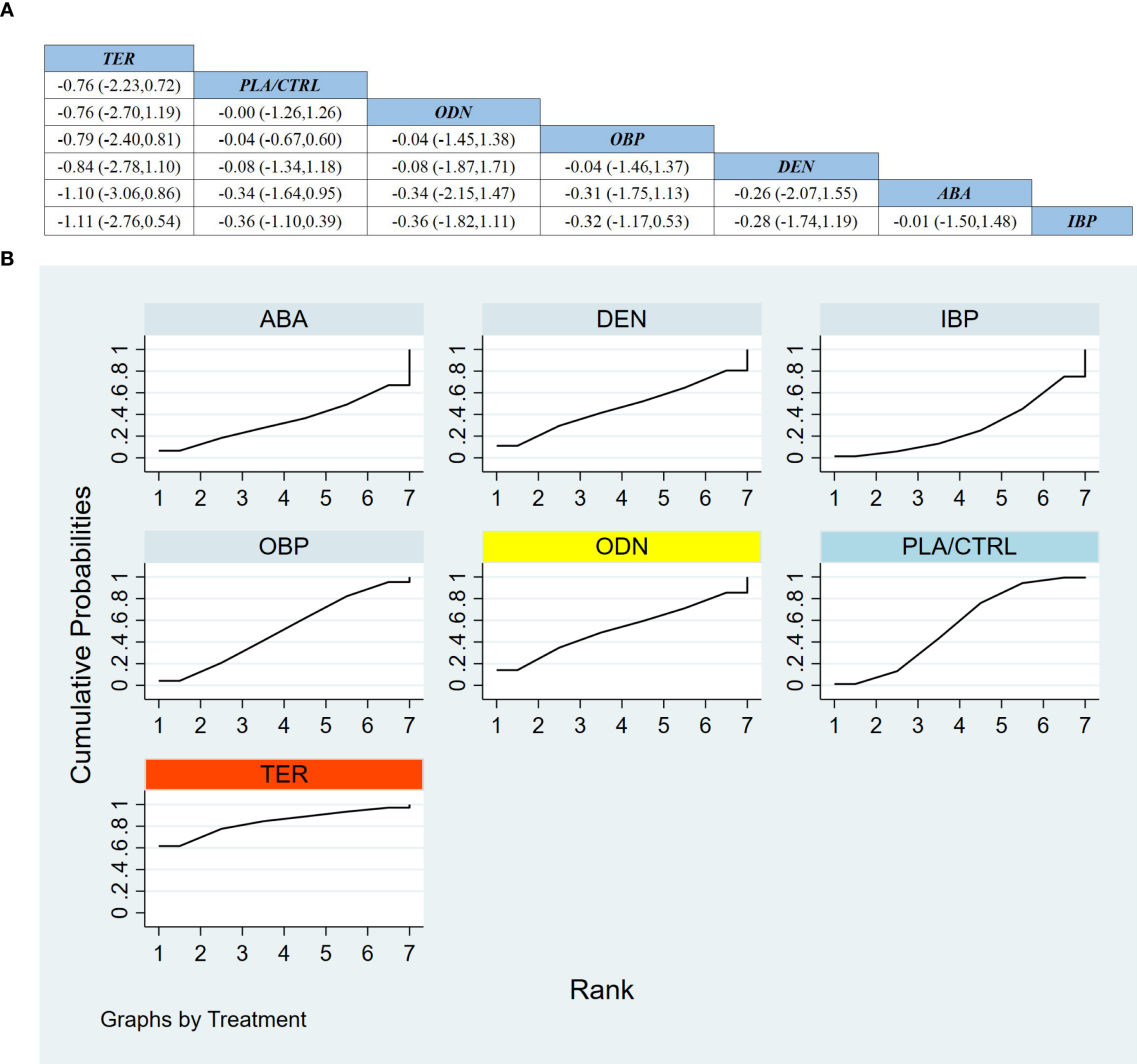
**(a)** The results of League table for all adverse events. **(b)** Ranking the probability of all adverse events.

Across 11 RCTs including 3,667 participants, we compared the incidence of any AEs across treatments. Overall, the network meta-analysis did not identify statistically significant differences in the risk of any AEs between most interventions, including teriparatide, and placebo/control or other active treatments, and many comparisons were associated with wide confidence intervals. Detailed comparisons are shown in **Figure 7a**.

Based on SUCRA (**Figure 7b**), the probability ranking for safety (any AEs) from highest to lowest was: TER (SUCRA 83.9%), PLA/CTRL (SUCRA 54.6%), ODN (SUCRA 52.2%), OBP (SUCRA 50.9%), DEN (SUCRA 46.6%), ABA (SUCRA 34.2%), and IBP (SUCRA 27.6%). However, these rankings should be interpreted with great caution, because SUCRA reflects relative ranking probabilities rather than definitive evidence of superior safety, and most comparisons were imprecise with uncertainty intervals that did not support clear clinical differentiation among treatments.

#### 3.3.2 Serious adverse events

**Figure 8:**
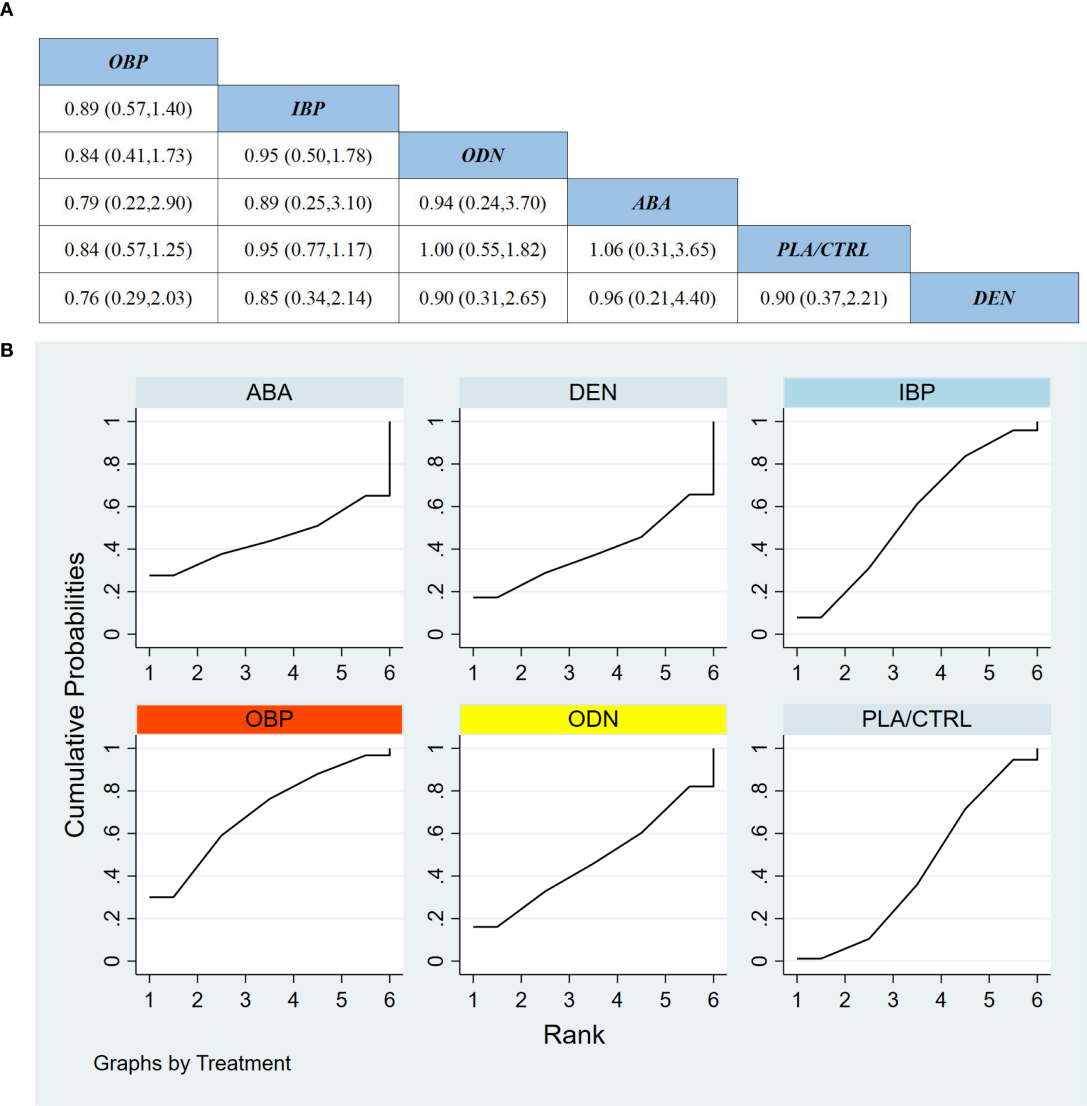
**(a)** The results of League table for serious adverse events. **(b)** Ranking the probability of serious adverse events.

Across 9 RCTs including 3,292 participants, we compared SAEs across treatments (TER was not included due to insufficient data). Overall, the network meta-analysis did not identify statistically significant differences in SAE risk between most interventions and placebo/control or between active treatments, and the available estimates were generally imprecise. Detailed comparisons are shown in **Figure 8a**.

Based on SUCRA (**Figure 8b**), the probability ranking for safety (SAEs) from highest to lowest was: OBP (SUCRA 70.0%), IBP (SUCRA 55.9%), ODN (SUCRA 47.4%), ABA (SUCRA 45.0%), PLA/CTRL (SUCRA 42.7%), and DEN (SUCRA 38.9%). However, these rankings should not be interpreted as evidence of definitive safety superiority, because the SAE network was limited in size, several comparisons had wide uncertainty, and TER was not included due to insufficient data.

## 4 Discussion

In this network meta-analysis (NMA), we synthesized randomized controlled trial (RCT) evidence in men with primary osteoporosis to compare the relative efficacy and safety of OBP, IBP, ABA, DEN, TER, and ODN, and ranked competing treatments using SUCRA. Overall, treatment effects were not fully consistent across skeletal sites: osteoanabolic therapies (ABA and TER) yielded larger gains in lumbar spine BMD (LS-BMD), whereas OBP and ABA showed more pronounced improvements in total hip BMD (TH-BMD); for femoral neck BMD (FN-BMD), statistically significant benefits versus PLA/CTRL were observed for TER and ABA. For safety outcomes, most comparisons for AEs and SAEs had wide confidence intervals, indicating limited power to differentiate safety profiles across drugs; thus, SUCRA-based rankings should be interpreted cautiously in conjunction with effect estimates and their precision.

From a mechanistic perspective, these findings are biologically plausible. The lumbar spine is enriched in trabecular bone and exhibits higher remodeling activity and may therefore be more responsive to bone-forming therapies. Both ABA and TER stimulate bone formation via PTH1R-related signaling, and their larger gains in LS-BMD versus PLA/CTRL, as well as superiority to OBP, align with established evidence that osteoanabolic agents have marked effects on vertebral bone mass. However, the difference between ABA and TER was not clearly established, suggesting that available evidence in men remains insufficient to determine their relative superiority. In clinical practice, treatment selection should therefore consider patient characteristics, accessibility, adherence, and risk management rather than relying solely on ranking differences. In addition, real-world decision-making must account for substantial patient heterogeneity, including differences in baseline fracture risk, age, prior fracture history, renal function, and comorbidity burden. Treatment sequencing may also influence the overall benefit-risk profile, particularly when anabolic and antiresorptive therapies are used in different clinical contexts or in sequential strategies. Accordingly, the present findings should be interpreted as comparative evidence to inform, rather than replace, individualized treatment decisions.

For hip-related outcomes, OBP, ABA, and TER significantly increased TH-BMD, and OBP showed a greater gain than TER. Improvements in hip BMD may depend more on sustained suppression of bone resorption and longer-term stabilization of remodeling balance. Given the mature antiresorptive mechanism and extensive clinical experience with bisphosphonates, a stable benefit in hip BMD is clinically plausible. ABA also produced significant gains in TH-BMD, suggesting that its impact on hip BMD in men may be meaningful; nevertheless, more direct comparisons and longer follow-up are needed to clarify whether these BMD gains translate into reductions in fracture endpoints.

For FN-BMD, TER and ABA significantly improved outcomes versus PLA/CTRL, whereas DEN, ODN, and IBP showed estimates favoring benefit but with wide uncertainty crossing the null. Because the femoral neck is a key site associated with hip fracture risk, FN-BMD improvement is clinically relevant. However, the largely non-significant comparisons among active agents indicate that current evidence is insufficient to conclude that any one active treatment is definitively superior for FN-BMD. A more robust interpretation is that evidence supporting FN-BMD gains is clearer for TER and ABA, while effects of DEN, ODN, and IBP remain imprecisely estimated and warrant further study. Although several treatment comparisons showed statistically significant improvements in BMD, the clinical magnitude of these changes should be interpreted with caution. In osteoporosis management, fracture prevention remains the most clinically meaningful goal, whereas BMD serves primarily as a surrogate marker of treatment response. Therefore, even when differences in LS-BMD, TH-BMD, or FN-BMD are statistically significant, they should not be assumed to translate directly into proportional reductions in absolute fracture risk. The clinical relevance of the observed BMD gains is best understood as supportive evidence of skeletal response rather than definitive proof of comparative fracture benefit.

Although SUCRA rankings provide an intuitive summary of relative ranking probabilities across multiple interventions, they do not represent definitive evidence of treatment superiority and should not be interpreted as a basis for clinical recommendation in isolation. In the present study, several interventions were associated with wide uncertainty intervals and limited direct comparative evidence, meaning that ranking positions may be unstable and potentially misleading if considered without reference to the underlying effect estimates. Therefore, clinical interpretation should prioritize the magnitude and precision of treatment effects, the consistency of evidence across skeletal sites and outcomes, and the broader clinical context, while SUCRA should be regarded only as supportive comparative information.

Regarding safety, the present network meta-analysis did not demonstrate statistically significant differences in the risk of overall AEs or SAEs for most interventions compared with placebo/control or with one another, and most estimates were accompanied by wide confidence intervals. Therefore, the safety findings should not be interpreted as establishing clear clinical superiority of one treatment over another. In particular, although SUCRA provides an intuitive ranking framework, a higher SUCRA value does not mean that a treatment is definitively “safer,” especially when the underlying comparisons are sparse or imprecise. This is particularly important for agents such as teriparatide, where a relatively high ranking for any AEs could otherwise be misread as evidence of greater safety than placebo/control, which is not supported by the present data.

In addition, the included trials did not consistently report drug-specific adverse outcomes of major clinical concern, such as atypical femoral fractures, osteonecrosis of the jaw, cardiovascular events, or stroke. As a result, the safety analyses should be regarded as broad summaries of overall reported AEs/SAEs rather than definitive comparative assessments of clinically critical adverse-event profiles.

ODN warrants special consideration. As a selective cathepsin K inhibitor, ODN may improve BMD by suppressing bone resorption, and its relatively favorable ranking for some BMD outcomes is therefore biologically plausible. However, its clinical development was discontinued following safety concerns, including stroke-related signals observed in the phase III Long-Term Odanacatib Fracture Trial (LOFT). Accordingly, although ODN may show potential BMD benefits within the present network, its current clinical applicability is highly limited. Its inclusion in this study should therefore be understood primarily in an academic and methodological context, providing historical comparative evidence within the network rather than supporting its use as a current therapeutic option. ODN-related findings should be interpreted with particular caution in light of unresolved long-term safety concerns, limited clinical endpoint evidence, and the absence of current clinical use.

This study has several strengths: (1) a focus on male primary osteoporosis, an area with relatively limited evidence but substantial clinical importance; (2) integration of direct and indirect evidence via NMA to provide comparative estimates despite limited head-to-head trials; and (3) assessment of both efficacy (multi-site BMD) and safety (AEs/SAEs) to enhance clinical relevance. However, limitations should be acknowledged. First, incomplete reporting of randomization, allocation concealment, blinding, and outcome reporting led to frequent ratings of unclear risk of bias across the included trials. Although an unclear rating does not necessarily indicate a high risk of bias, it reflects insufficient methodological transparency and reduces confidence in the internal validity of the evidence base. As a result, the pooled estimates should be interpreted with appropriate caution, because bias related to study design or reporting may have influenced the observed effects. Second, several comparisons exhibited wide confidence intervals, indicating limited precision and potentially unstable SUCRA rankings. In addition, the evidence network was largely centered on placebo/control comparisons, with relatively few direct head-to-head trials among active treatments. As a result, several comparative estimates between active interventions depended heavily on indirect evidence, which reduces robustness and increases reliance on the assumptions of transitivity and consistency. Although no significant local inconsistency was detected, the limited number of active-comparator trials means that indirect comparisons may still be more susceptible to bias and should therefore be interpreted cautiously. Residual clinical heterogeneity may also have influenced the pooled estimates. Although we assessed transitivity clinically and methodologically and preferentially extracted outcome data from time points closest to 12 months to improve comparability, formal subgroup or meta-regression analyses based on variables such as age, baseline BMD, or study duration were not performed. This was primarily because the available male-specific RCT evidence was sparse, several treatment nodes were informed by only a limited number of studies, and potential effect modifiers were not reported consistently across trials. Therefore, some between-study variability may remain insufficiently explained. Third, efficacy analyses were largely based on follow-up durations close to 12 months, limiting evaluation of long-term maintenance of benefit and delayed adverse events. In addition, the eligibility criteria were restricted to men with primary osteoporosis, and studies involving secondary osteoporosis or combination therapies were excluded to improve clinical comparability and reduce methodological heterogeneity. However, this restriction may limit the generalizability of the findings, because these populations and treatment strategies are common in real-world clinical practice. Therefore, the present results should be interpreted primarily in the context of pharmacological monotherapy for primary male osteoporosis. Fourth, although BMD is a clinically important surrogate marker, fracture reduction remains the gold-standard endpoint for evaluating osteoporosis therapies. Therefore, the present findings should be interpreted primarily as comparative evidence on BMD response rather than definitive evidence of comparative anti-fracture efficacy. Some included studies also required imputation of missing variance data when standard deviations could not be derived from the reported statistics. Although a relatively large imputed SD was used to reduce the statistical influence of those studies and avoid overstating precision, this approach may still introduce additional uncertainty and should be considered when interpreting the pooled estimates. Bisphosphonates were analyzed at the class level (oral bisphosphonates and intravenous bisphosphonates) rather than as individual agents. This grouping strategy was adopted to preserve network connectivity and analytical feasibility in the context of limited randomized evidence and sparse direct comparisons in men with primary osteoporosis. Although these drugs share a common antiresorptive mechanism, individual bisphosphonates may differ in potency, pharmacokinetics, dosing schedules, and tolerability. Therefore, this class-based analysis may have obscured potentially relevant within-class differences in efficacy and safety. Finally, safety outcomes were analyzed using aggregate AE/SAE endpoints, which are inherently heterogeneous and may obscure clinically important drug-specific adverse-event patterns. Teriparatide was not included in the SAE network because of limited available data, and this incomplete network may have reduced the stability and comparability of indirect safety estimates and may also have influenced the relative ranking of the remaining treatments. Therefore, SAE-based comparisons and rankings should be interpreted with particular caution.

Future research should prioritize high-quality RCTs in men, particularly head-to-head comparisons of key therapies and longer follow-up, with consistent reporting of fracture endpoints and drug-specific adverse events. For agents with novel mechanisms (e.g., cathepsin K inhibitors), systematic evaluation of serious cardiovascular and cerebrovascular risks and risk stratification is essential to support robust benefit–risk assessments.

In summary, among men with primary osteoporosis, treatment effects on BMD vary by skeletal site: ABA and TER show greater gains in LS-BMD, OBP demonstrates robust improvements in TH-BMD, and evidence for FN-BMD improvement is clearer for TER and ABA. Safety evidence remains limited, and SUCRA rankings should be interpreted cautiously alongside effect estimates and their precision. These findings provide a comprehensive comparative evidence base to support individualized pharmacotherapy selection in male osteoporosis, while highlighting the need for additional high-quality long-term studies to strengthen the evidence framework.

## 5 Conclusion

Based on RCT evidence, this network meta-analysis systematically compared the relative efficacy and safety of oral bisphosphonates (OBP), intravenous bisphosphonates (IBP), abaloparatide (ABA), denosumab (DEN), teriparatide (TER), and odanacatib (ODN) in men with primary osteoporosis. Treatment effects on BMD differed by skeletal site: ABA and TER yielded more pronounced improvements in lumbar spine BMD, OBP and ABA showed robust gains in total hip BMD, and evidence for femoral neck BMD improvement was clearer for TER and ABA compared with placebo/control. However, because the included RCTs provided limited direct fracture endpoint data, the extent to which these BMD improvements translate into fracture risk reduction remains uncertain. Therefore, the present findings should be interpreted as comparative evidence on BMD response and overall safety signals rather than definitive evidence of anti-fracture superiority.

For safety outcomes, most comparisons for any adverse events (AEs) and serious adverse events (SAEs) were imprecise with wide confidence intervals, and the network meta-analysis did not demonstrate clear statistically significant differences between most interventions. Therefore, SUCRA rankings should be interpreted cautiously in conjunction with effect estimates and their precision and should not be used as a standalone basis for clinical decision-making. Given its discontinuation from clinical development and unresolved safety concerns, ODN should not be interpreted as a current therapeutic option in clinical practice; its inclusion in the present study is primarily of academic and methodological relevance. Further high-quality RCTs and long-term follow-up studies in men are warranted, particularly those directly comparing key therapies and reporting fracture endpoints and drug-specific adverse events, to better define long-term benefit–risk profiles.

## References

[1] Subarajan P Arceo-Mendoza RM Camacho PM . Postmenopausal osteoporosis: a review of latest guidelines. Endocrinol Metab Clinics North America. (2024) 53:497–512. doi: 10.1016/j.ecl.2024.08.008 39448132

[2] Arceo-Mendoza RM Camacho PM . Postmenopausal osteoporosis: latest guidelines. Endocrinol Metab Clinics North America. (2021) 50:167–78. doi: 10.1016/j.ecl.2021.03.009 34023036

[3] Johnston CB Dagar M . Osteoporosis in older adults. Med Clinics North America. (2020) 104:873–84. doi: 10.1016/j.mcna.2020.06.004. PMID: 32773051

[4] Fischer V Haffner-Luntzer M . Interaction between bone and immune cells: implications for postmenopausal osteoporosis. Semin Cell Dev Biol. (2022) 123:14–21. doi: 10.1016/j.semcdb.2021.05.014. PMID: 34024716

[5] Bandeira L Silva BC Bilezikian JP . Male osteoporosis. Arch Endocrinol Metab. (2022) 66:739–47. doi: 10.20945/2359-3997000000563. PMID: 36382763 PMC10118818

[6] Muñoz M Robinson K Shibli-Rahhal A . Bone health and osteoporosis prevention and treatment. Clin Obstetrics Gynecology. (2020) 63:770–87. doi: 10.1097/GRF.0000000000000572 33017332

[7] Cortet B Guañabens N Brandi ML Siggelkow H . Similarities and differences between European guidelines for the management of postmenopausal osteoporosis. Arch Osteoporosis. (2024) 19:84. doi: 10.1007/s11657-024-01441-z. PMID: 39235671 PMC11377466

[8] Gao S Zhao Y . Quality of life in postmenopausal women with osteoporosis: a systematic review and meta-analysis. Qual Life Research: Int J Qual Life Aspects Treatment Care Rehabil. (2023) 32:1551–65. doi: 10.1007/s11136-022-03281-1. PMID: 36383282

[9] Słupski W Jawień P Nowak B . Botanicals in postmenopausal osteoporosis. Nutrients. (2021) 13(5):1609. doi: 10.3390/nu13051609 34064936 PMC8151026

[10] Pietschmann P Kerschan-Schindl K . Osteoporosis: gender-specific aspects. Wiener Medizinische Wochenschrift (1946). (2004) 154:411–5. doi: 10.1007/s10354-004-0100-1. PMID: 15552228

[11] Wadhwa H Wu JY Lee JS Zygourakis CC . Anabolic and antiresorptive osteoporosis treatment: trends, costs, and sequence in a commercially insured population, 2003-2021. JBMR Plus. (2023) 7:e10800. doi: 10.1002/jbm4.10800. PMID: 37808398 PMC10556263

[12] Cosman F . Anabolic therapy and optimal treatment sequences for patients with osteoporosis at high risk for fracture. Endocrine Practice: Off J Am Coll Endocrinol Am Assoc Clin Endocrinologists. (2020) 26:777–86. doi: 10.4158/ep-2019-0596. PMID: 33471647

[13] Haas AV LeBoff MS . Osteoanabolic agents for osteoporosis. J Endocr Soc. (2018) 2:922–32. doi: 10.1210/js.2018-00118. PMID: 30087947 PMC6065487

[14] Cosman F Nieves JW Dempster DW . Treatment sequence matters: anabolic and antiresorptive therapy for osteoporosis. J Bone Mineral Research: Off J Am Soc For Bone Mineral Res. (2017) 32:198–202. doi: 10.1002/jbmr.3051. PMID: 27925287

[15] Cosman F . Anabolic and antiresorptive therapy for osteoporosis: combination and sequential approaches. Curr Osteoporosis Rep. (2014) 12:385–95. doi: 10.1007/s11914-014-0237-9. PMID: 25341476

[16] Vilaca T Eastell R . Antiresorptive versus anabolic therapy in managing osteoporosis in people with type 1 and type 2 diabetes. JBMR Plus. (2023) 7:e10838. doi: 10.1002/jbm4.10838. PMID: 38025034 PMC10652175

[17] Elsman EBM Mokkink LB Terwee CB Beaton D Gagnier JJ Tricco AC . Guideline for reporting systematic reviews of outcome measurement instruments (OMIs): PRISMA-COSMIN for OMIs 2024. J Patient-Reported Outcomes. (2024) 8:64. doi: 10.1016/j.jclinepi.2024.111422. PMID: 38977535 PMC11231111

[18] Pu HY Chen Q Huang K Wei P . Correlation between forearm bone mineral density measured by dual energy X-ray absorptiometry and Hounsfield units value measured by CT in lumbar spine. Z Fur Orthopadie und Unfallchirurgie. (2024) 162:247–53. doi: 10.1055/a-1984-0466. PMID: 36720241

[19] Davidson S Vecellio A Flagstad I Holton K Bruzina A Lender P . Discrepancy between DXA and CT-based assessment of spine bone mineral density. Spine Deformity. (2023) 11:677–83. doi: 10.1007/s43390-023-00646-5. PMID: 36735159

[20] Davenport A . Differences in prevalence of reduced and low bone mineral density between lumbar spine and femoral neck in peritoneal dialysis patients using dual-energy X-ray absorptiometry (DXA). Peritoneal Dialysis International: J Int Soc For Peritoneal Dialysis. (2023) 43:334–8. doi: 10.1177/08968608221146867. PMID: 36627766

[21] Shojaa M von Stengel S Kohl M Schoene D Kemmler W . Effects of dynamic resistance exercise on bone mineral density in postmenopausal women: a systematic review and meta-analysis with special emphasis on exercise parameters. Osteoporosis International: A J Established as Result Cooperation Between Eur Foundation For Osteoporosis Natl Osteoporosis Foundation USA. (2020) 31:1427–44. doi: 10.1007/s00198-020-05441-w. PMID: 32399891 PMC7360540

[22] Kemmler W Shojaa M Kohl M von Stengel S . Effects of different types of exercise on bone mineral density in postmenopausal women: a systematic review and meta-analysis. Calcif Tissue Int. (2020) 107:409–39. doi: 10.1007/s00223-020-00744-w. PMID: 32785775 PMC7546993

[23] Bouxsein ML Eastell R Lui LY Wu LA de Papp AE Grauer A . Change in bone density and reduction in fracture risk: a meta-regression of published trials. J Bone Mineral Research: Off J Am Soc For Bone Mineral Res. (2019) 34:632–42. doi: 10.1002/jbmr.3641. PMID: 30674078

[24] Pereira TV Jüni P Saadat P Xing D Yao L Bobos P . Viscosupplementation for knee osteoarthritis: systematic review and meta-analysis. BMJ (Clinical Res Ed). (2022) 378:e069722. doi: 10.1136/bmj-2022-069722. PMID: 36333100 PMC9258606

[25] Wu Q Taboureau O Audouze K . Development of an adverse drug event network to predict drug toxicity. Curr Res Toxicol. (2020) 1:48–55. doi: 10.1016/j.crtox.2020.06.001. PMID: 34345836 PMC8320634

[26] Cashin AG Folly T Bagg MK Wewege MA Jones MD Ferraro MC . Efficacy, acceptability, and safety of muscle relaxants for adults with non-specific low back pain: systematic review and meta-analysis. BMJ (Clinical Res Ed). (2021) 374:n1446. doi: 10.1136/bmj.n1446. PMID: 34233900 PMC8262447

[27] Cashin AG Furlong BM Kamper SJ De Carvalho D MaChado LA Davidson SR . Analgesic effects of non-surgical and non-interventional treatments for low back pain: a systematic review and meta-analysis of placebo-controlled randomised trials. BMJ Evidence-Based Med. (2025) 3:222–32. doi: 10.1136/bmjebm-2024-112974. PMID: 40101974

[28] Agarwal A Rochwerg B Sevransky JE . 21st century evidence: randomized controlled trials versus systematic reviews and meta-analyses. Crit Care Med. (2021) 49:2001–2. doi: 10.1097/ccm.0000000000005343. PMID: 34582418 PMC8654119

[29] Seidler AL Hunter KE Cheyne S Berlin JA Ghersi D Askie LM . Prospective meta-analyses and Cochrane’s role in embracing next-generation methodologies. Cochrane Database Systematic Rev. (2020) 10:Ed000145. doi: 10.1002/14651858.ed000145. PMID: 33284462 PMC9414316

[30] Moher D Schulz KF Altman D . The CONSORT statement: revised recommendations for improving the quality of reports of parallel-group randomized trials. JAMA. (2001) 285:1987–91. doi: 10.1001/jama.285.15.1987. PMID: 11308435

[31] Sehmbi H Retter S Shah UJ Nguyen D Martin J Uppal V . Epidemiological, methodological, and statistical characteristics of network meta-analysis in anaesthesia: a systematic review. Br J Anaesthesia. (2023) 130:272–86. doi: 10.1016/j.bja.2022.08.042. PMID: 36404140

[32] Béliveau A Boyne DJ Slater J Brenner D Arora P . BUGSnet: an R package to facilitate the conduct and reporting of Bayesian network meta-analyses. BMC Med Res Methodol. (2019) 19:196. doi: 10.1186/s12874-019-0829-2 31640567 PMC6805536

[33] Yang F Wang H Zou J Li X Jin X Cao Y . Assessing the methodological and reporting quality of network meta-analyses in Chinese medicine. Medicine. (2018) 97:e13052. doi: 10.1097/md.0000000000013052. PMID: 30461607 PMC6392701

[34] Eastell R Brown JP Adler RA Lewiecki EM Binkley N Orwoll ES . Bone turnover markers predict changes in bone mineral density in men treated with abaloparatide: results from the abaloparatide for the treatment of men with osteoporosis (ATOM) study. J Bone Mineral Research: Off J Am Soc For Bone Mineral Res. (2025) 40:315–22. doi: 10.1093/jbmr/zjaf003. PMID: 39791502 PMC11909733

[35] Matsumoto T Sone T Soen S Tanaka S Yamashita A Inoue T . Abaloparatide increases lumbar spine and hip BMD in Japanese patients with osteoporosis: the phase 3 ACTIVE-J study. J Clin Endocrinol Metab. (2022) 107:e4222–31. doi: 10.1210/clinem/dgac486. PMID: 35977548 PMC9516124

[36] Czerwinski E Cardona J Plebanski R Recknor C Vokes T Saag KG . The efficacy and safety of abaloparatide-SC in men with osteoporosis: a randomized clinical trial. J Bone Mineral Research: Off J Am Soc For Bone Mineral Res. (2022) 37:2435–42. doi: 10.1002/jbmr.4719. PMID: 36190391 PMC10091818

[37] Qi YJ Wang WY Sun WL Pan QY . Comparative efficacy and safety of alendronate and teriparatide in bone loss reduction and prevention of vertebral fracture in osteoporotic Chinese patients. Trop J Pharm Res. (2021) 20:2199–204. doi: 10.4314/tjpr.v20i10.26

[38] Binkley N Orwoll E Chapurlat R Langdahl BL Scott BB Giezek H . Randomized, controlled trial to assess the safety and efficacy of odanacatib in the treatment of men with osteoporosis. Osteoporosis International: A J Established as Result Cooperation Between Eur Foundation For Osteoporosis Natl Osteoporosis Foundation USA. (2021) 32:173–84. doi: 10.1007/s00198-020-05701-9. PMID: 33200257

[39] Nakamura T Shiraki M Fukunaga M Tomomitsu T Santora AC Tsai R . Effect of the cathepsin K inhibitor odanacatib administered once weekly on bone mineral density in Japanese patients with osteoporosis--a double-blind, randomized, dose-finding study. Osteoporosis International: A J Established as Result Cooperation Between Eur Foundation For Osteoporosis Natl Osteoporosis Foundation USA. (2014) 25:367–76. doi: 10.1007/s00198-013-2398-2. PMID: 23716037

[40] Walker MD Cusano NE Sliney J Jr Romano M Zhang C McMahon DJ . Combination therapy with risedronate and teriparatide in male osteoporosis. Endocrine. (2013) 44:237–46. doi: 10.1007/s12020-012-9819-4. PMID: 23099796

[41] Orwoll E Teglbjærg CS Langdahl BL Chapurlat R Czerwinski E Kendler DL . A randomized, placebo-controlled study of the effects of denosumab for the treatment of men with low bone mineral density. J Clin Endocrinol Metab. (2012) 97:3161–69. doi: 10.1210/jc.2012-1569. PMID: 22723310

[42] Boonen S Reginster JY Kaufman JM Lippuner K Zanchetta J Langdahl B . Fracture risk and zoledronic acid therapy in men with osteoporosis. N Engl J Med. (2012) 367:1714–23. doi: 10.1056/nejmoa1204061. PMID: 23113482

[43] Boonen S Orwoll E Magaziner J Colón-Emeric CS Adachi JD Bucci-Rechtweg C . Once-yearly zoledronic acid in older men compared with women with recent hip fracture. J Am Geriatrics Soc. (2011) 59:2084–90. doi: 10.1111/j.1532-5415.2011.03666.x. PMID: 22091563

[44] Orwoll ES Miller PD Adachi JD Brown J Adler RA Kendler D . Efficacy and safety of a once-yearly i.v. infusion of zoledronic acid 5 mg versus a once-weekly 70-mg oral alendronate in the treatment of male osteoporosis: a randomized, multicenter, double-blind, active-controlled study. J Bone Mineral Research: Off J Am Soc For Bone Mineral Res. (2010) 25:2239–50. doi: 10.1002/jbmr.119. PMID: 20499357

[45] Orwoll ES Binkley NC Lewiecki EM Gruntmanis U Fries MA Dasic G . Efficacy and safety of monthly ibandronate in men with low bone density. Bone. (2010) 46:970–6. doi: 10.1016/j.bone.2009.12.034. PMID: 20060082

[46] Hwang JS Liou MJ Ho C Lin JD Huang YY Wang CJ . The effects of weekly alendronate therapy in Taiwanese males with osteoporosis. J Bone Miner Metab. (2010) 28:328–33. doi: 10.1007/s00774-009-0136-9. PMID: 20012918

[47] Ringe JD Farahmand P Faber H Dorst A . Sustained efficacy of risedronate in men with primary and secondary osteoporosis: results of a 2-year study. Rheumatol Int. (2009) 29:311–5. doi: 10.1007/s00296-008-0689-2. PMID: 18762944

[48] Boonen S Orwoll ES Wenderoth D Stoner KJ Eusebio R Delmas PD . Once-weekly risedronate in men with osteoporosis: results of a 2-year, placebo-controlled, double-blind, multicenter study. J Bone Mineral Research: Off J Am Soc For Bone Mineral Res. (2009) 24:719–25. doi: 10.1359/jbmr.081214. PMID: 19049326

[49] Kaufman JM Orwoll E Goemaere S San Martin J Hossain A Dalsky GP . Teriparatide effects on vertebral fractures and bone mineral density in men with osteoporosis: treatment and discontinuation of therapy. Osteoporosis International: A J Established as Result Cooperation Between Eur Foundation For Osteoporosis Natl Osteoporosis Foundation USA. (2005) 16:510–6. doi: 10.1007/s00198-004-1713-3. PMID: 15322742

[50] Ringe JD Dorst A Faber H Ibach K . Alendronate treatment of established primary osteoporosis in men: 3-year results of a prospective, comparative, two-arm study. Rheumatol Int. (2004) 24:110–3. doi: 10.1007/s00296-003-0388-y. PMID: 13680141

[51] Miller PD Schnitzer T Emkey R Orwoll E Rosen C Ettinger M . Weekly oral alendronic acid in male osteoporosis. Clin Drug Invest. (2004) 24:333–41. doi: 10.2165/00044011-200424060-00003. PMID: 17516720

[52] Orwoll ES Scheele WH Paul S Adami S Syversen U Diez-Perez A . The effect of teriparatide [human parathyroid hormone (1-34)] therapy on bone density in men with osteoporosis. J Bone Mineral Research: Off J Am Soc For Bone Mineral Res. (2003) 18:9–17. doi: 10.1016/b978-0-12-412650-3.50025-9. PMID: 12510800

[53] Gonnelli S Cepollaro C Montagnani A Bruni D Caffarelli C Breschi M . Alendronate treatment in men with primary osteoporosis: a three-year longitudinal study. Calcif Tissue Int. (2003) 73:133–9. doi: 10.1007/s00223-002-1085-7. PMID: 14565594

[54] Orwoll E Ettinger M Weiss S Miller P Kendler D Graham J . Alendronate for the treatment of osteoporosis in men. N Engl J Med. (2000) 343:604–10. doi: 10.1056/nejm200008313430902. PMID: 10979796

