# Supplementary material for: Comparative efficacy and safety of odanacatib, abaloparatide, denosumab, teriparatide, and bisphosphonates for male osteoporosis: a systematic review and network meta-analysis
Source: Front Endocrinol (Lausanne). 2026 May 8;17:1836818. doi: 10.3389/fendo.2026.1836818 (PMC13193890; doi:10.3389/fendo.2026.1836818)

**Appendix.**

**Supplementary data**

**Search Strategy;**

**PUBMED （430）**

((((((((((((((((((((Osteoporosis[MeSH Terms]) OR (Osteoporoses[Title/Abstract])) OR (Osteoporosis, Age-Related[Title/Abstract])) OR (Osteoporosis, Age Related[Title/Abstract])) OR (Age-Related Osteoporosis[Title/Abstract])) OR (Age-Related Osteoporoses[Title/Abstract])) OR (Age Related Osteoporosis[Title/Abstract])) OR (Osteoporoses, Age-Related[Title/Abstract])) OR (Bone Loss, Age-Related[Title/Abstract])) OR (Age-Related Bone Loss[Title/Abstract])) OR (Age-Related Bone Losses[Title/Abstract])) OR (Bone Loss, Age Related[Title/Abstract])) OR (Bone Losses, Age-Related[Title/Abstract])) OR (Osteoporosis, Senile[Title/Abstract])) OR (Osteoporoses, Senile[Title/Abstract])) OR (Senile Osteoporoses[Title/Abstract])) OR (Senile Osteoporosis[Title/Abstract])) OR (Osteoporosis, Involutional[Title/Abstract])) AND (men[Title/Abstract]))) AND (((((((((((((((((((((((((((((((((Alendronate[MeSH Terms]) OR (Aminohydroxybutane Bisphosphonate[Title/Abstract])) OR (4-Amino-1-Hydroxybutylidene 1,1-Biphosphonate[Title/Abstract])) OR (MK-217[Title/Abstract])) OR (MK217[Title/Abstract])) OR (MK 217[Title/Abstract])) OR (Fosamax[Title/Abstract])) OR (Alendronate Sodium[Title/Abstract])) OR (Alendronate Monosodium Salt, Trihydrate[Title/Abstract])) OR (Risedronic Acid[MeSH Terms])) OR (Bisphosphonate Risedronate Sodium[Title/Abstract])) OR (Risedronate Sodium, Bisphosphonate[Title/Abstract])) OR (Sodium, Bisphosphonate Risedronate[Title/Abstract])) OR (Risedronate Sodium[Title/Abstract])) OR (Actonel[Title/Abstract])) OR (Risedronic Acid, Monosodium Salt[Title/Abstract])) OR (Risedronate[Title/Abstract])) OR (1-Hydroxy-2-(3-pyridyl)ethylidene diphosphonate[Title/Abstract])) OR (Atelvia[Title/Abstract])) OR (2-(3-pyridinyl)-1-hydroxyethylidene-bisphosphonate[Title/Abstract])) OR (2-(3-pyridinyl)-1-hydroxyethylidenebisphosphonate[Title/Abstract])) OR (Teriparatide[MeSH Terms])) OR (hPTH (1-34[Title/Abstract]))) OR (Human Parathyroid Hormone (1-34[Title/Abstract]))) OR (Parathar[Title/Abstract])) OR (Teriparatide Acetate[Title/Abstract])) OR (Forteo[Title/Abstract])) OR (Denosumab[MeSH Terms])) OR (AMG 162[Title/Abstract])) OR (Xgeva[Title/Abstract])) OR (Prolia[Title/Abstract]))) OR (Odanacatib))

**Cochrane Library （314）**

#1 MeSH descriptor: [Osteoporosis] explode all trees

#2 MeSH descriptor: [Men] explode all trees

#3 MeSH descriptor: [Male] explode all trees

#4 #2 or #3

#5 #1 and #4

#6 MeSH descriptor: [Alendronate] explode all trees

#7 MeSH descriptor: [Risedronic Acid] explode all trees

#8 MeSH descriptor: [Zoledronic Acid] explode all trees

#9 MeSH descriptor: [Denosumab] explode all trees

#10 MeSH descriptor: [Teriparatide] explode all trees

#11 abaloparatide

#12 Odanacatib

#13 #6 OR #7 OR #8 OR #9 OR #10 OR #11 OR #12

#14 #5 AND #13

**Web of science （1316）**

1: (((((((((((((((((TS=(Osteoporosis)) OR TS=(Osteoporoses)) OR TS=(Osteoporosis, Age-Related)) OR TS=(Osteoporosis, Age Related)) OR TS=(Age-Related Osteoporosis)) OR TS=(Age-Related Osteoporoses)) OR TS=(Age Related Osteoporosis)) OR TS=(Osteoporoses, Age-Related)) OR TS=(Bone Loss, Age-Related)) OR TS=(Age-Related Bone Loss)) OR TS=(Age-Related Bone Losses)) OR TS=(Bone Loss, Age Related)) OR TS=(Bone Losses, Age-Related)) OR TS=(Osteoporosis, Senile)) OR TS=(Osteoporoses, Senile)) OR TS=(Senile Osteoporoses)) OR TS=(Senile Osteoporosis)) OR TS=(Osteoporosis, Involutional)

2: TS=(men)

3: ((((((((((((((((((((((((((((((((TS=(Alendronate)) OR TS=(Aminohydroxybutane Bisphosphonate)) OR TS=(4-Amino-1-Hydroxybutylidene 1,1-Biphosphonate)) OR TS=(MK-217)) OR TS=(MK217)) OR TS=(MK 217)) OR TS=(Fosamax)) OR TS=(Alendronate Sodium)) OR TS=(Alendronate Monosodium Salt, Trihydrate)) OR TS=(Risedronic Acid)) OR TS=(Bisphosphonate Risedronate Sodium)) OR TS=(Risedronate Sodium, Bisphosphonate)) OR TS=(Sodium, Bisphosphonate Risedronate)) OR TS=(Risedronate Sodium)) OR TS=(Actonel)) OR TS=(Risedronic Acid, Monosodium Salt)) OR TS=(Risedronate)) OR TS=(1-Hydroxy-2-(3-pyridyl)ethylidene diphosphonate)) OR TS=(Atelvia)) OR TS=(2-(3-pyridinyl)-1-hydroxyethylidene-bisphosphonate)) OR TS=(2-(3-pyridinyl)-1-hydroxyethylidenebisphosphonate)) OR TS=(Teriparatide)) OR TS=(hPTH (1-34))) OR TS=(Human Parathyroid Hormone (1-34))) OR TS=(Parathar)) OR TS=(Teriparatide Acetate))) OR TS=(Forteo)) OR TS=(Denosumab)) OR TS=(AMG 162)) OR TS=(Xgeva)) OR TS=(Prolia)) OR TS=(Odanacatib)

4: #1 AND #2

5: #3 AND #4

|  | The Global inconsistency | ＞0.05 | DIC of Model of consistency | DIC of Model of inconsistency | The difference is  less than 5 |
| --- | --- | --- | --- | --- | --- |
| Lumbar spine BMD | 0.3184 | yes | 30.04183 | 33.00426 | yes |
| Femoral neck BMD | 0.4529 | yes | 19.28471 | 30.61892 | yes |
| Total hip BMD | 0.7617 | yes | 24.73855 | 27.94108 | yes |
| All adverse events | 0.9236 | yes | 21.61297 | 19.39144 | yes |
| Serious adverse events | 0.7015 | yes | 18.18736 | 14.90211 | yes |

**Supplementary Table 1.** Assessment of model fit. If the difference of DIC value in two modes is within 5, it means that the data is consistent. DIC, deviance information criterion.

**Supplementary Figure 1.** The [forest map](javascript:;) of all outcomes.

a)The results of [forest map](javascript:;) for Femoral neck BMD.


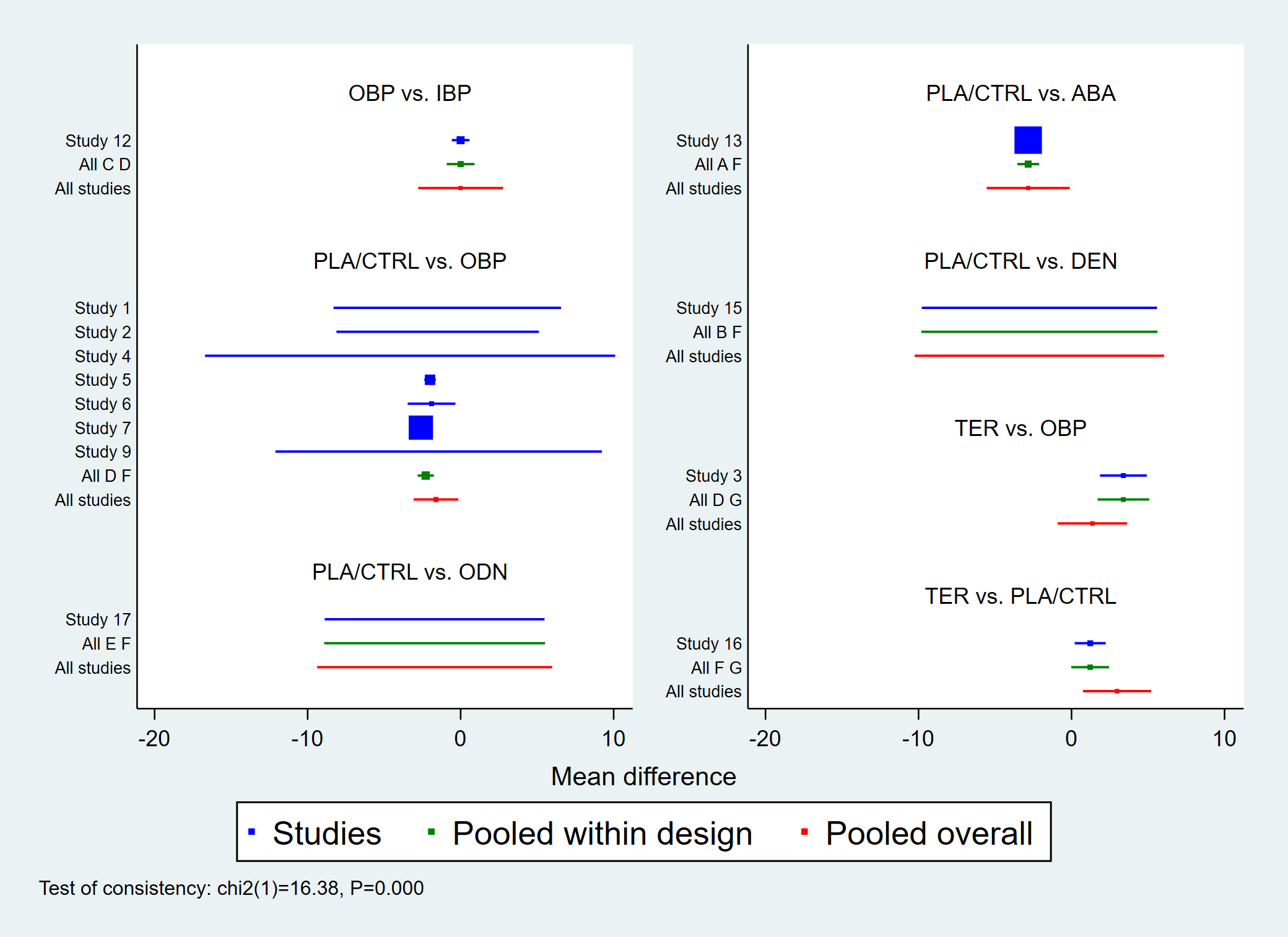


b) The results of [forest map](javascript:;) for Total hip BMD.


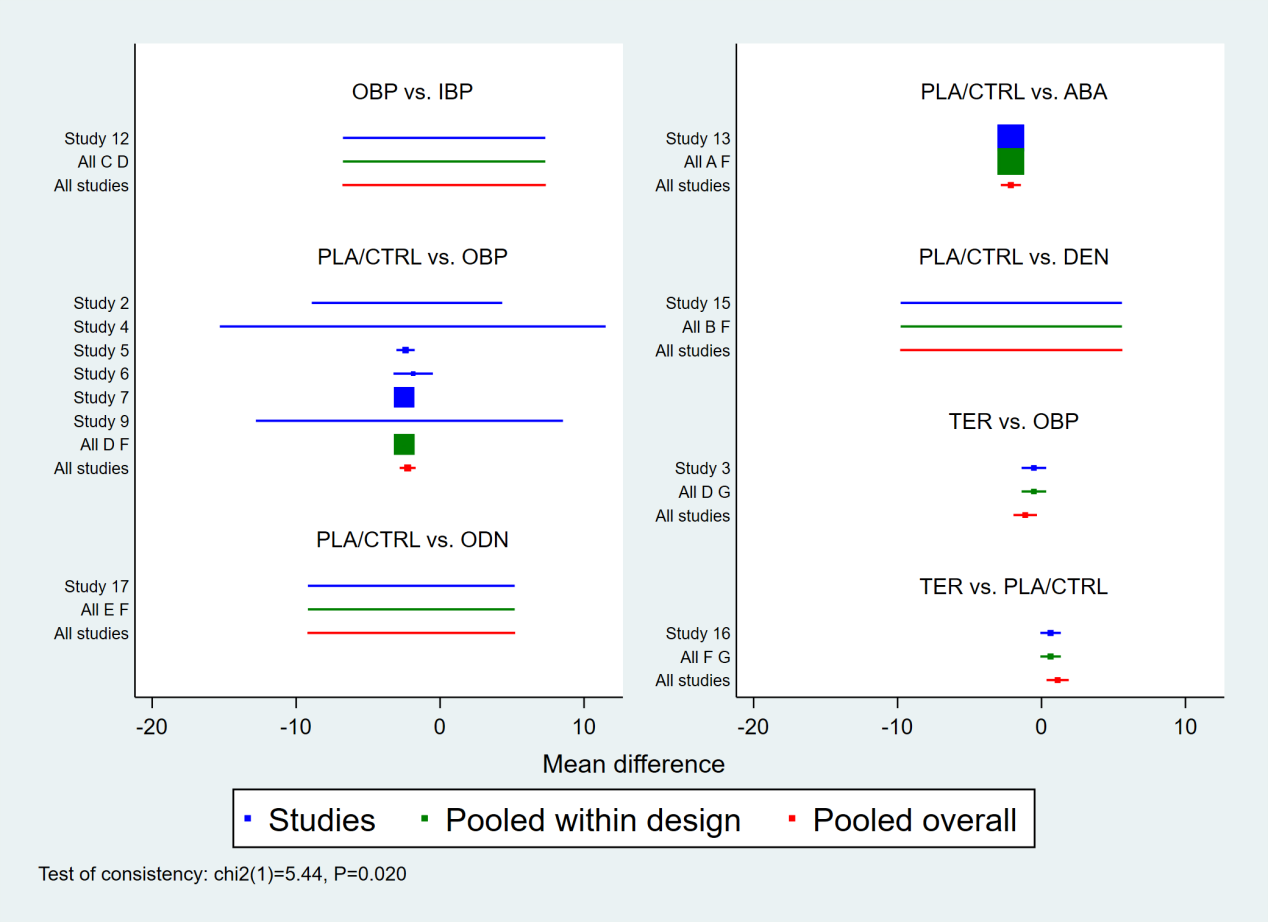


c) The results of [forest map](javascript:;) for Lumbar spine BMD.


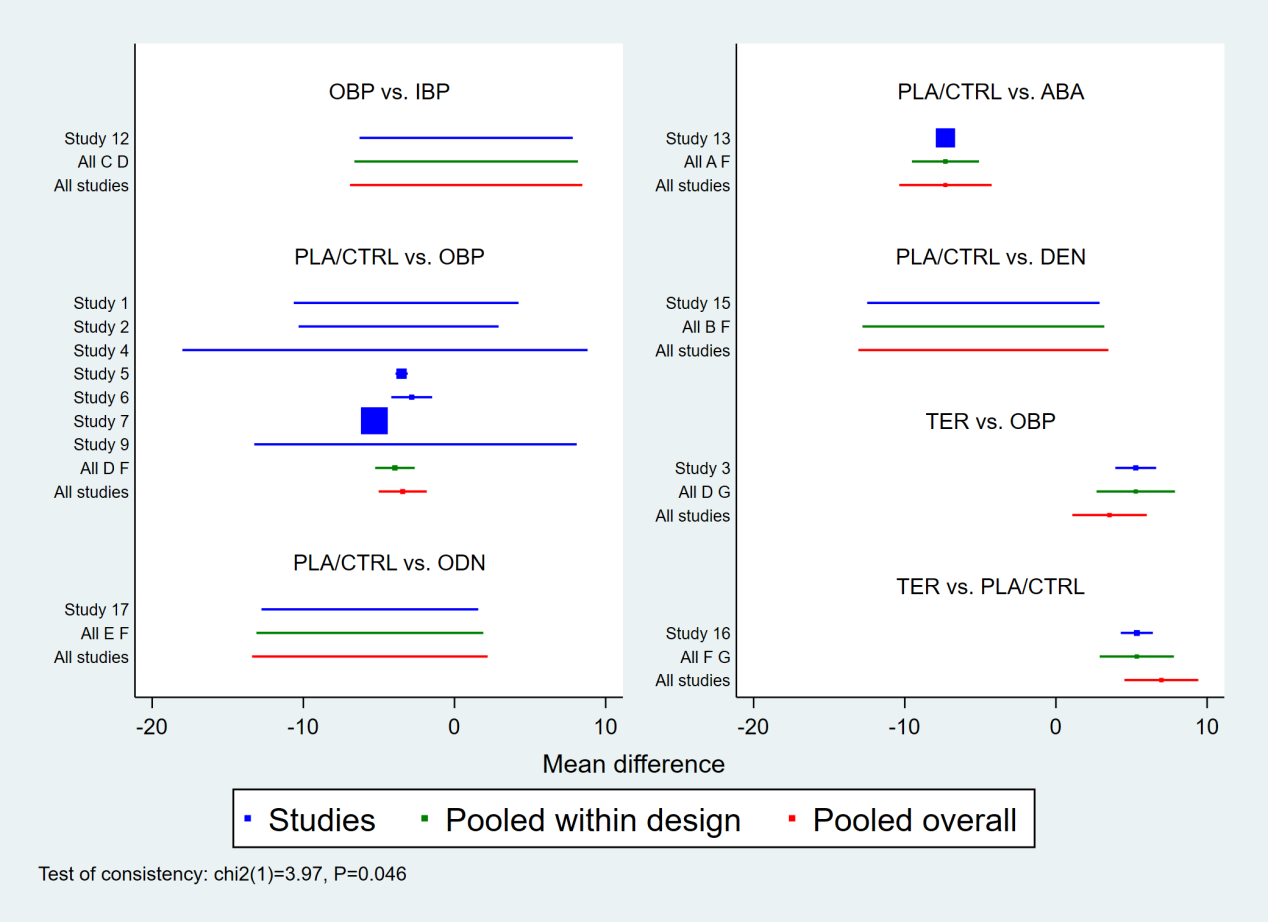


d) The results of [forest map](javascript:;) for All adverse events.


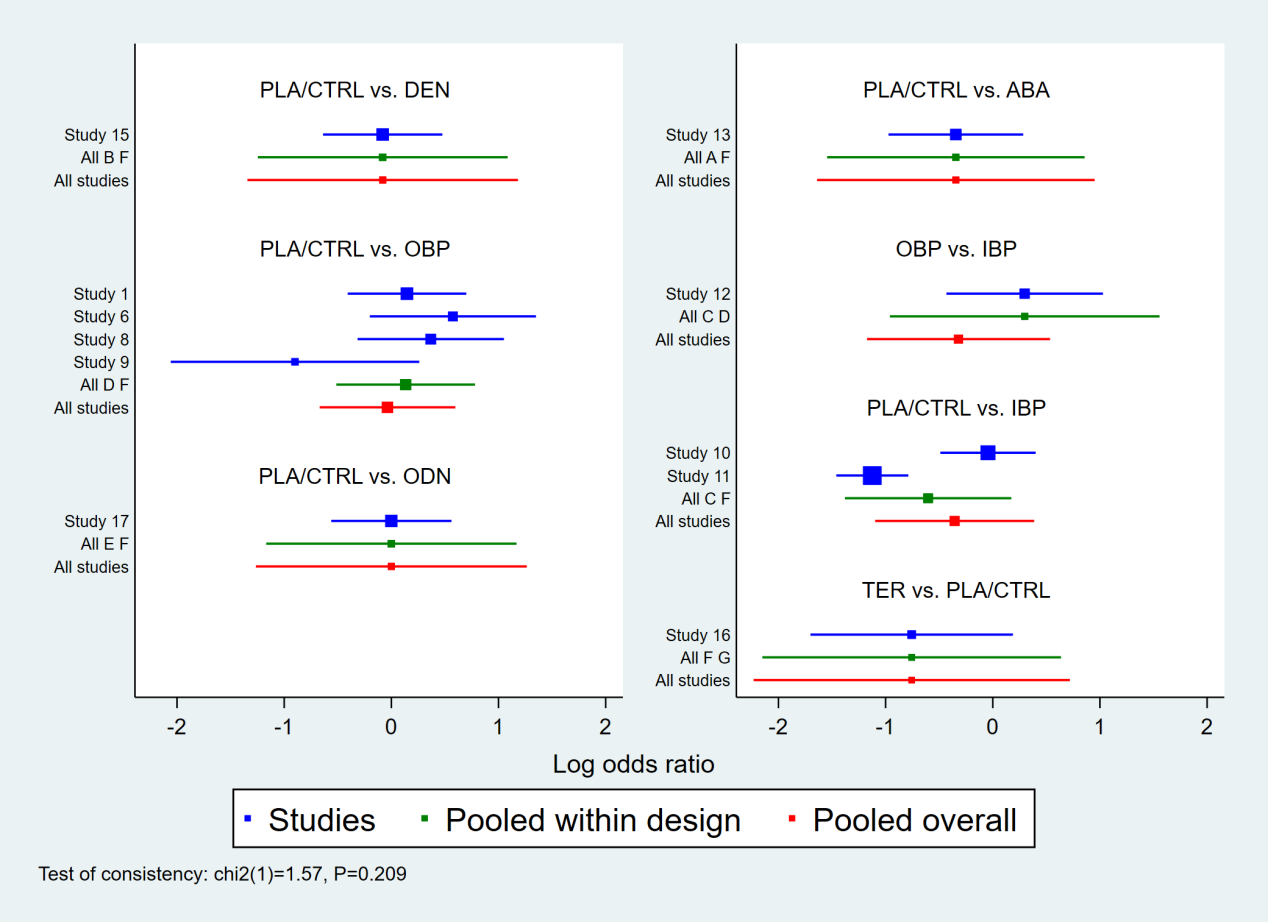


e) The results of [forest map](javascript:;) for Serious adverse events.


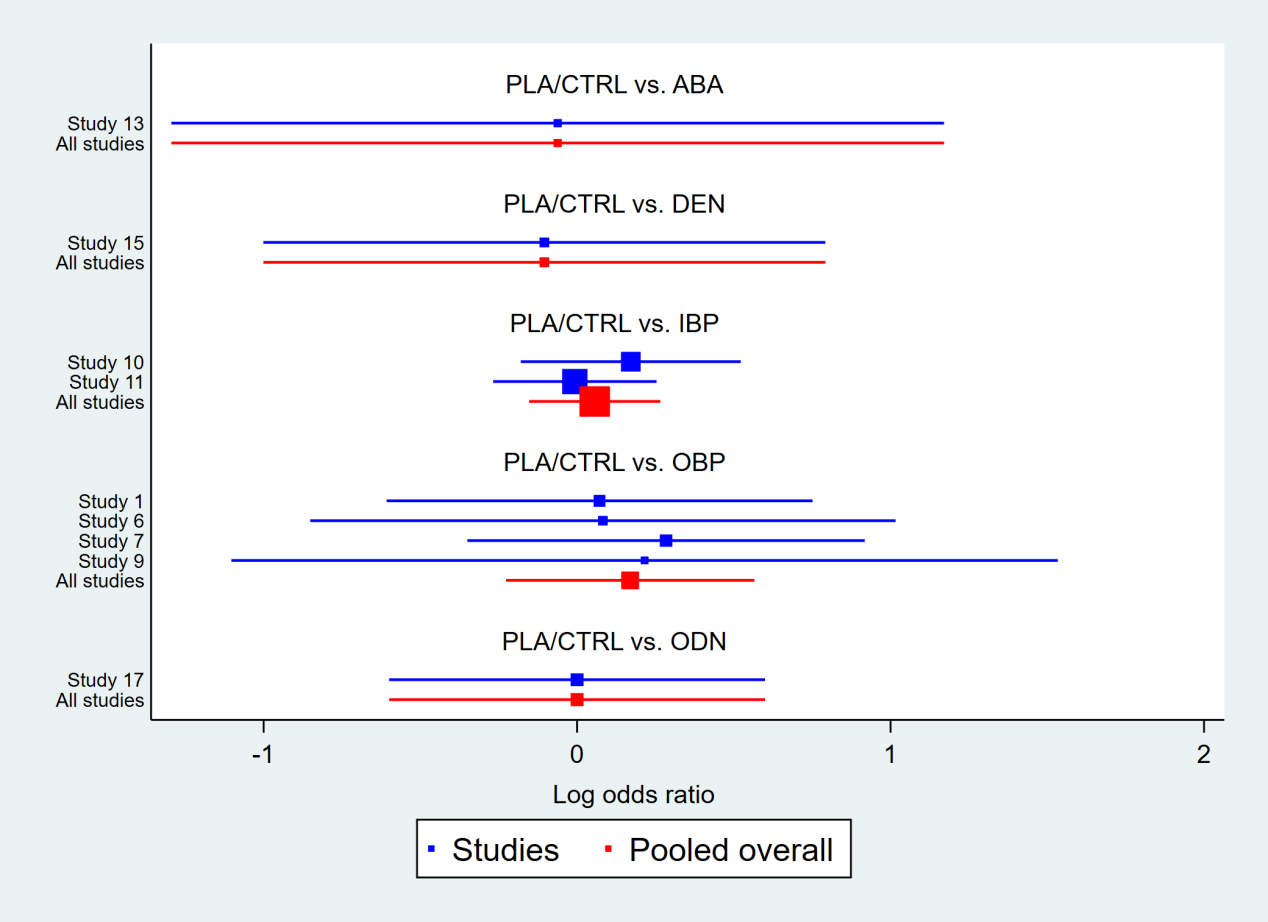


**Supplementary Figure 2.** The network plot of all outcomes.

1. Femoral neck BMD


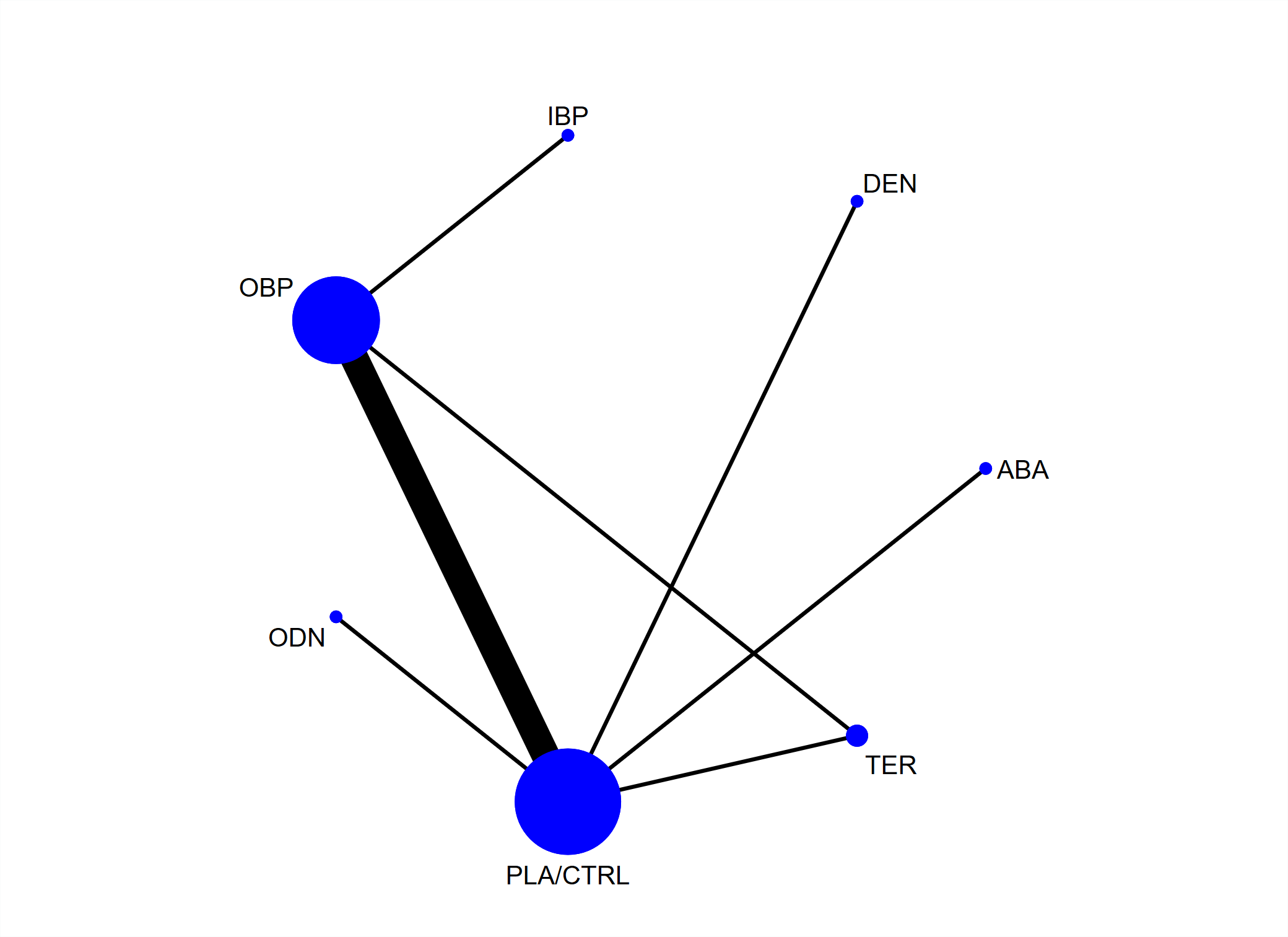


1. Total hip BMD


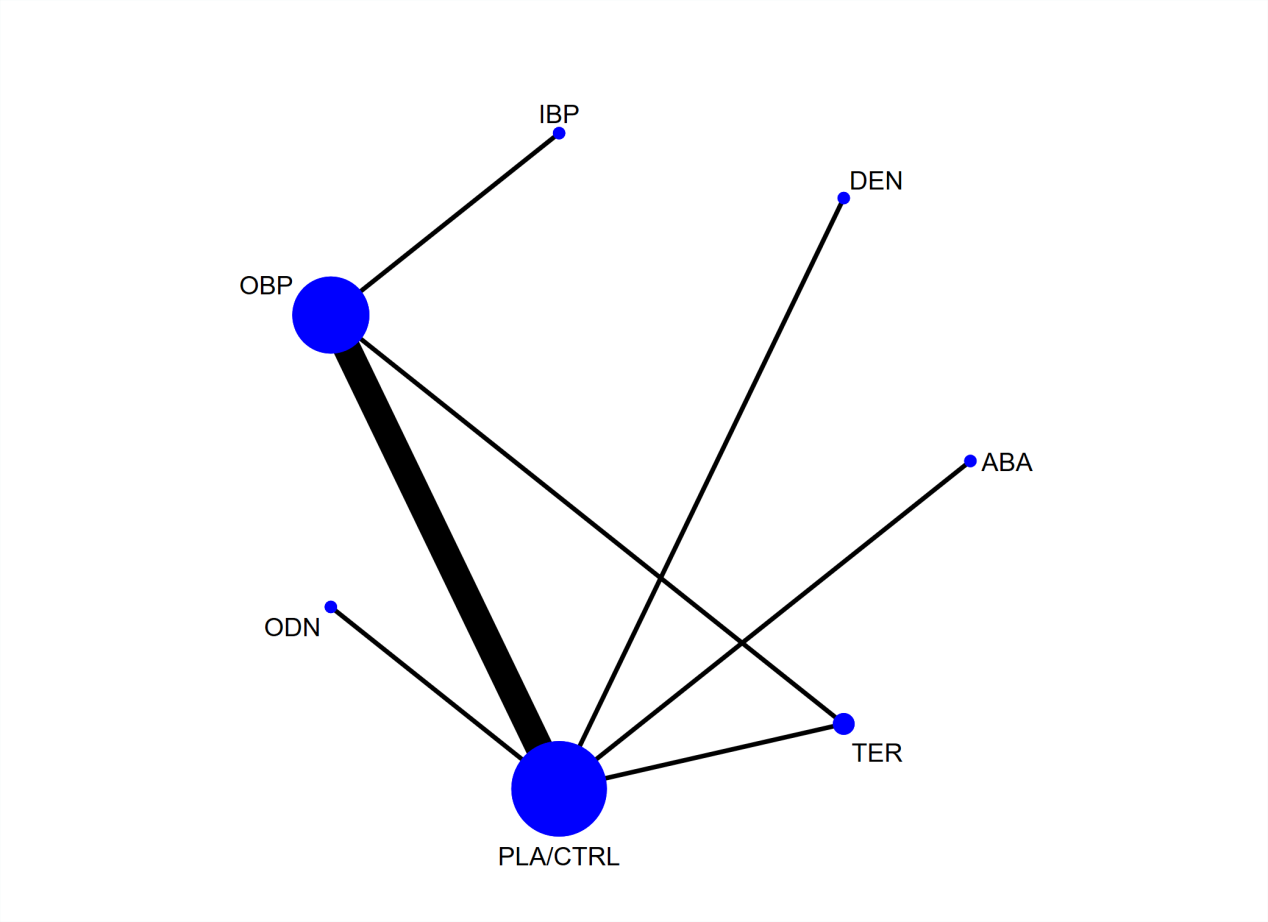


1. Lumbar spine BMD


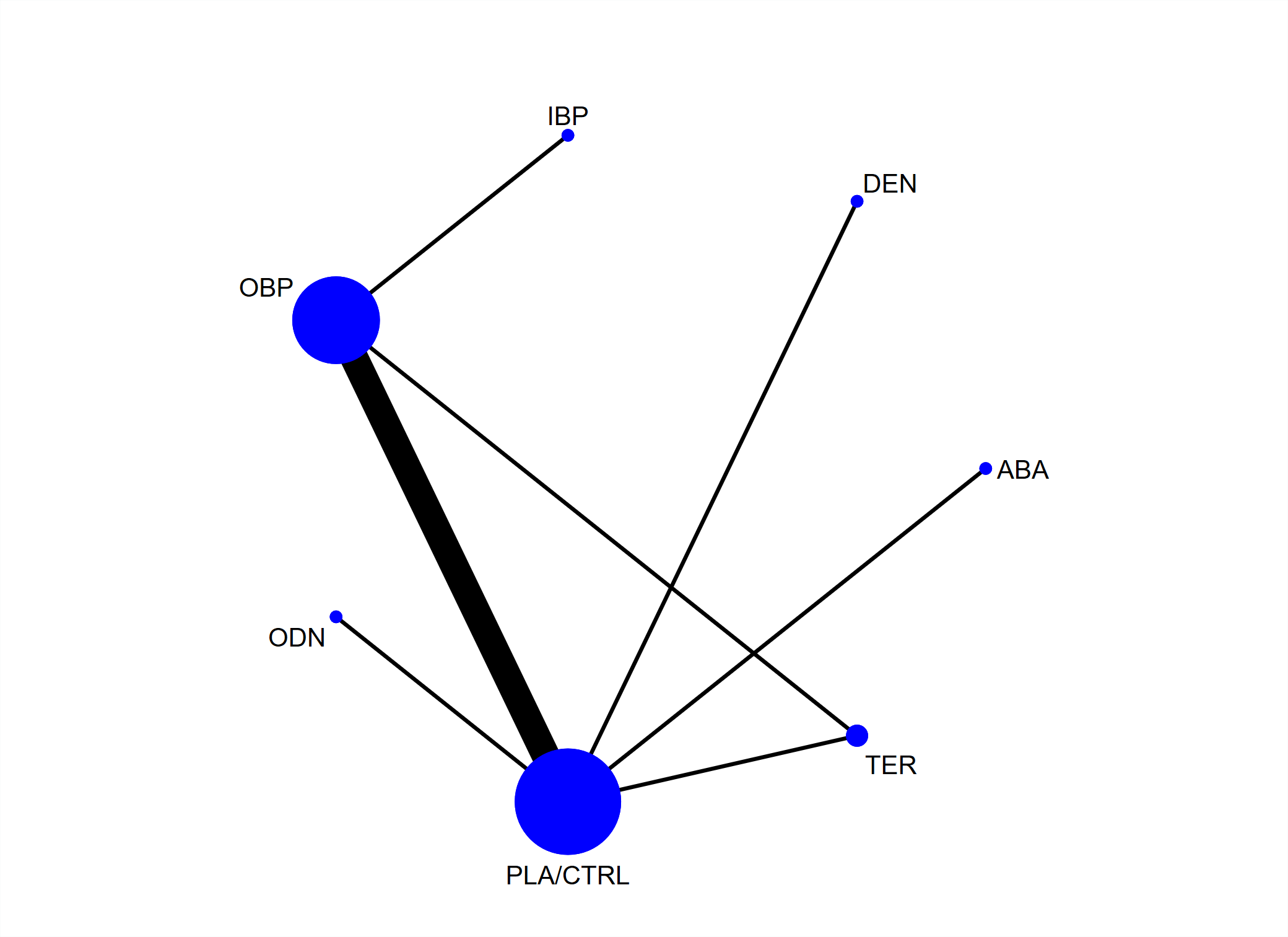


1. All adverse events


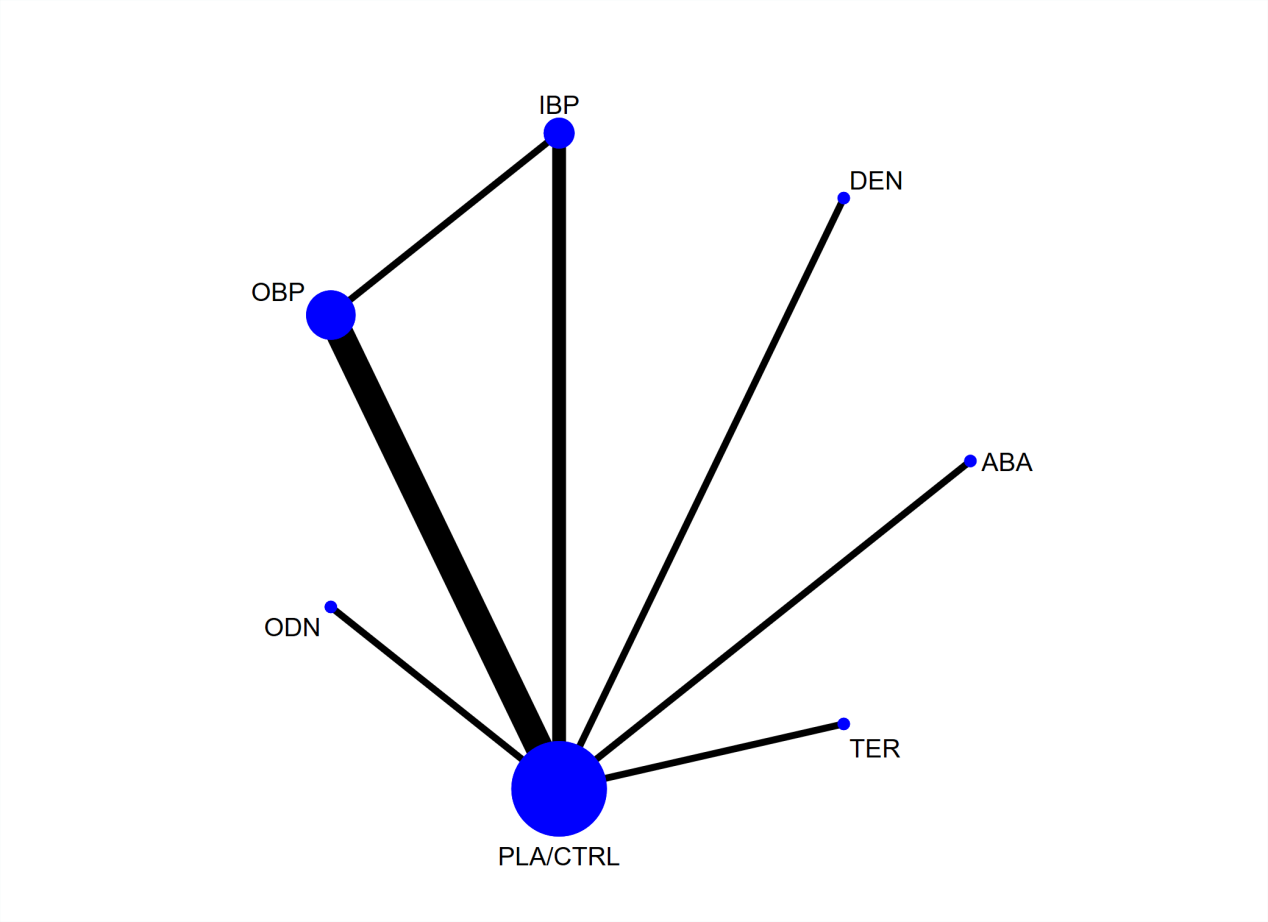


1. Serious adverse events


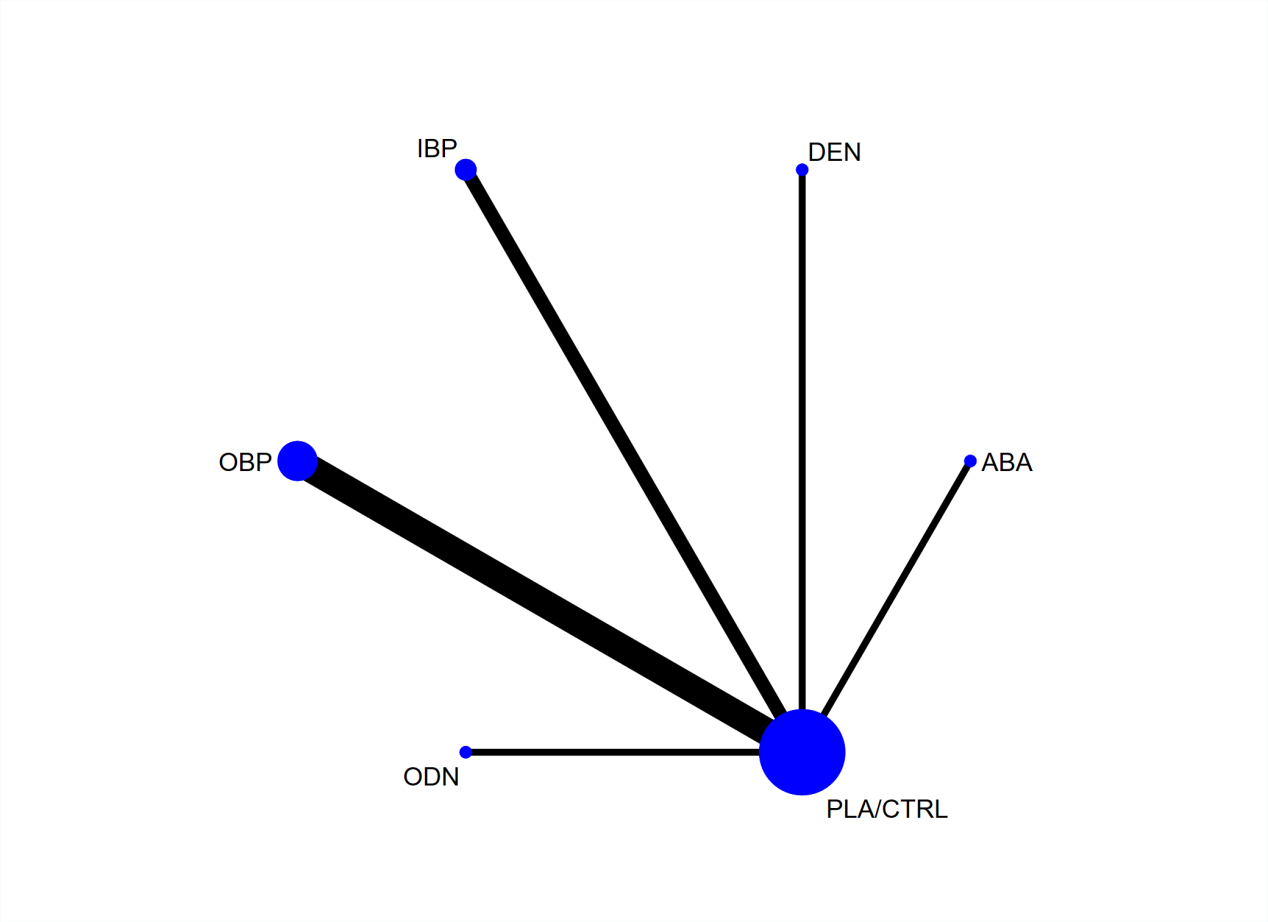


**Supplementary Figure 3.** The Funnel plot of all outcomes.

1. Femoral neck BMD


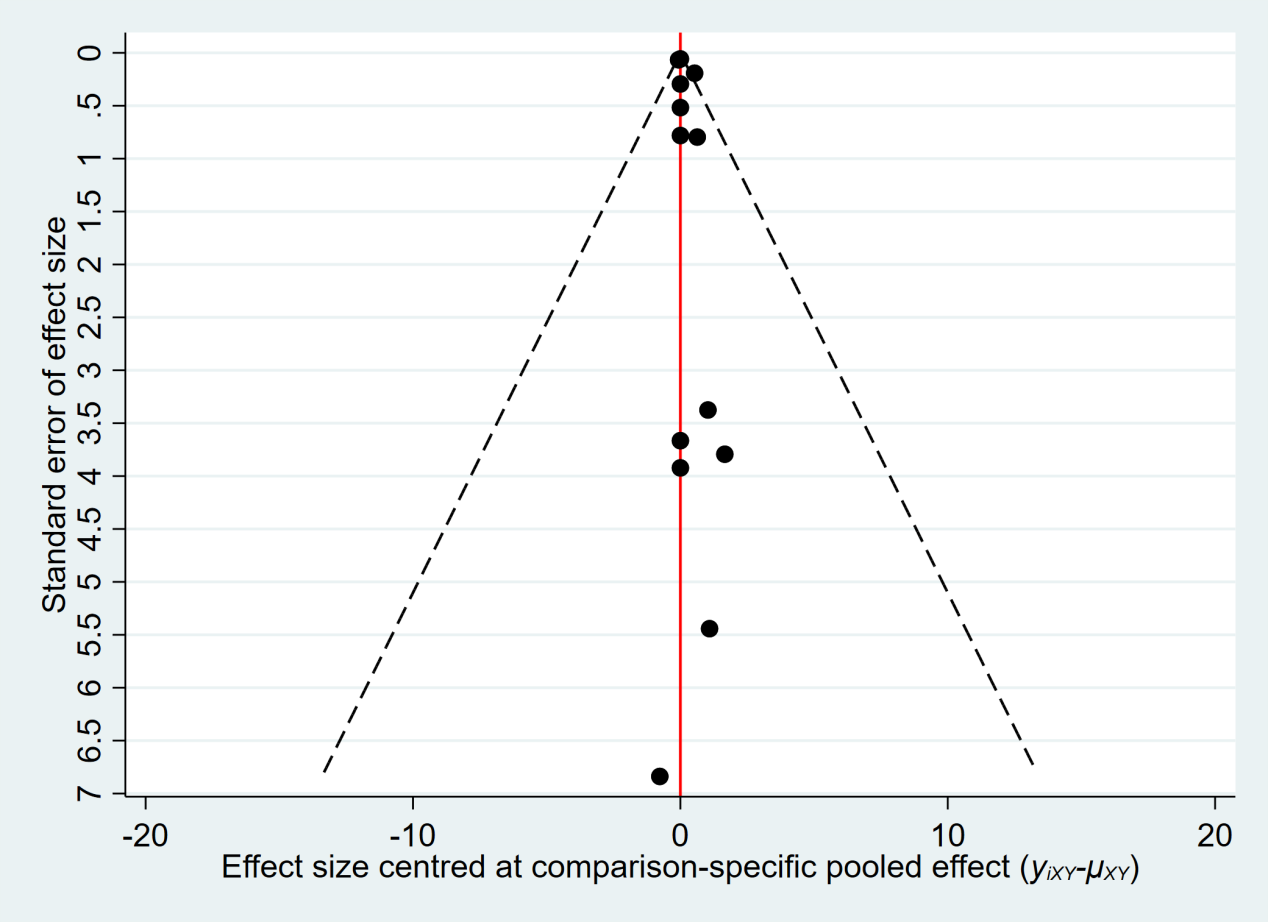


1. Total hip BMD


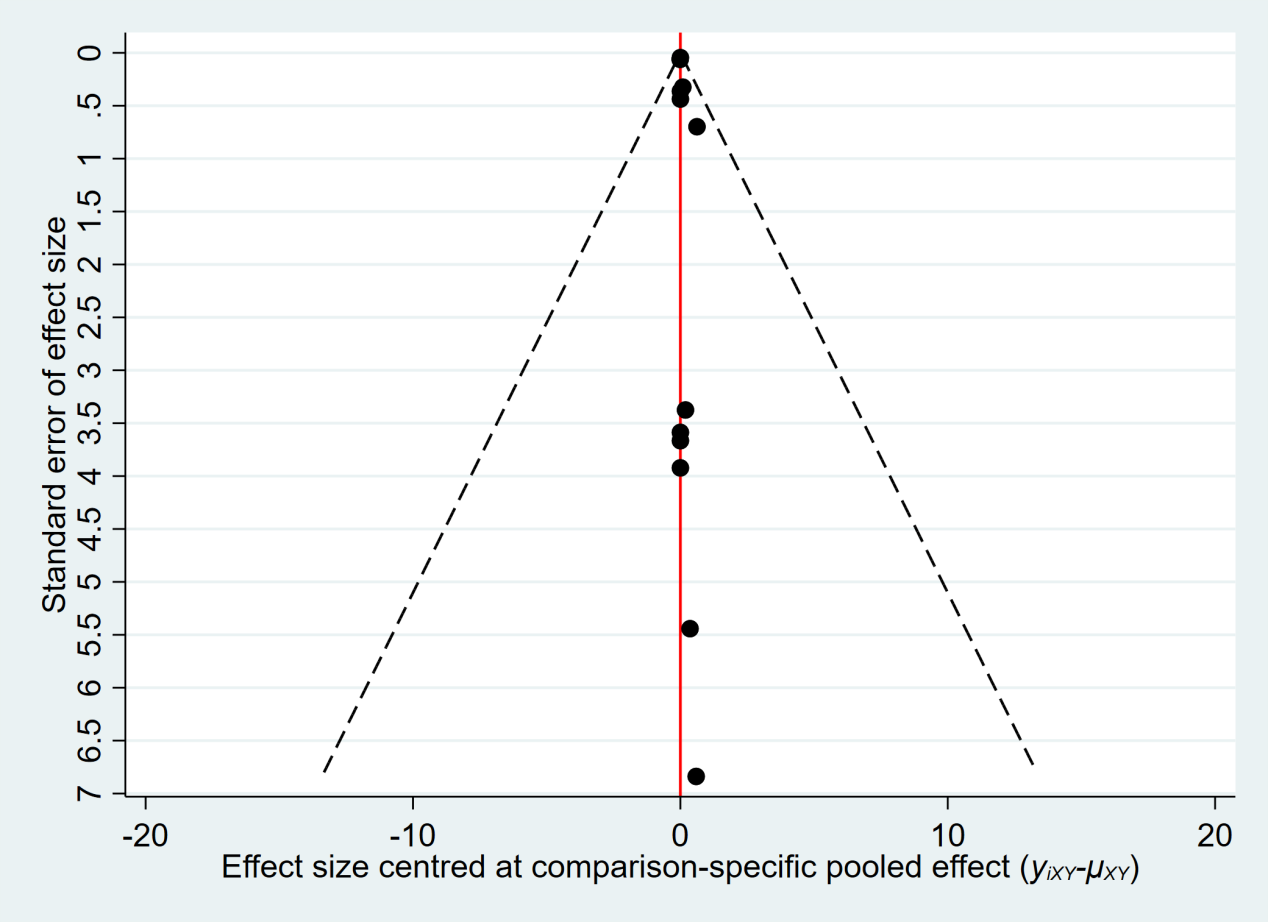


1. Lumbar spine BMD


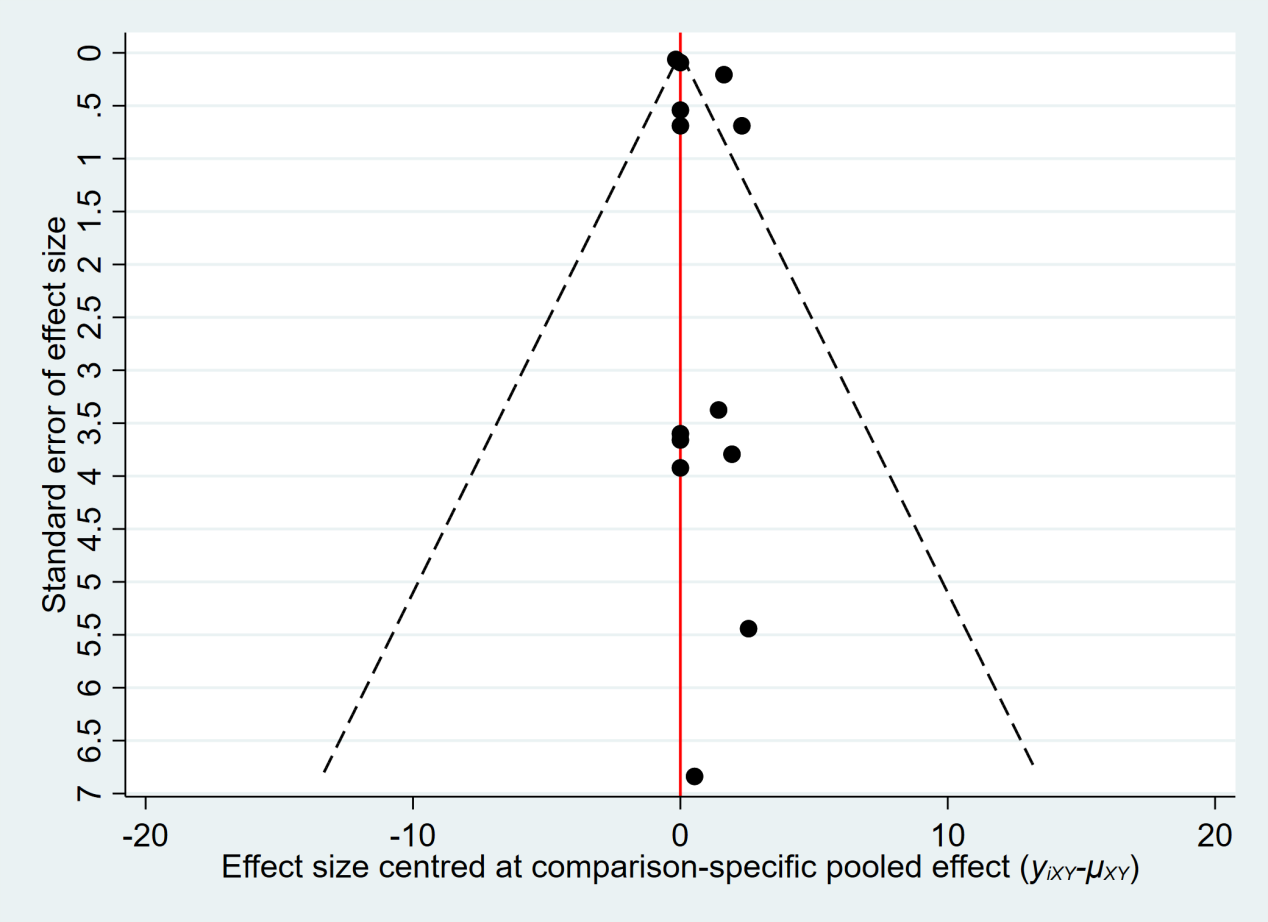


1. All adverse events


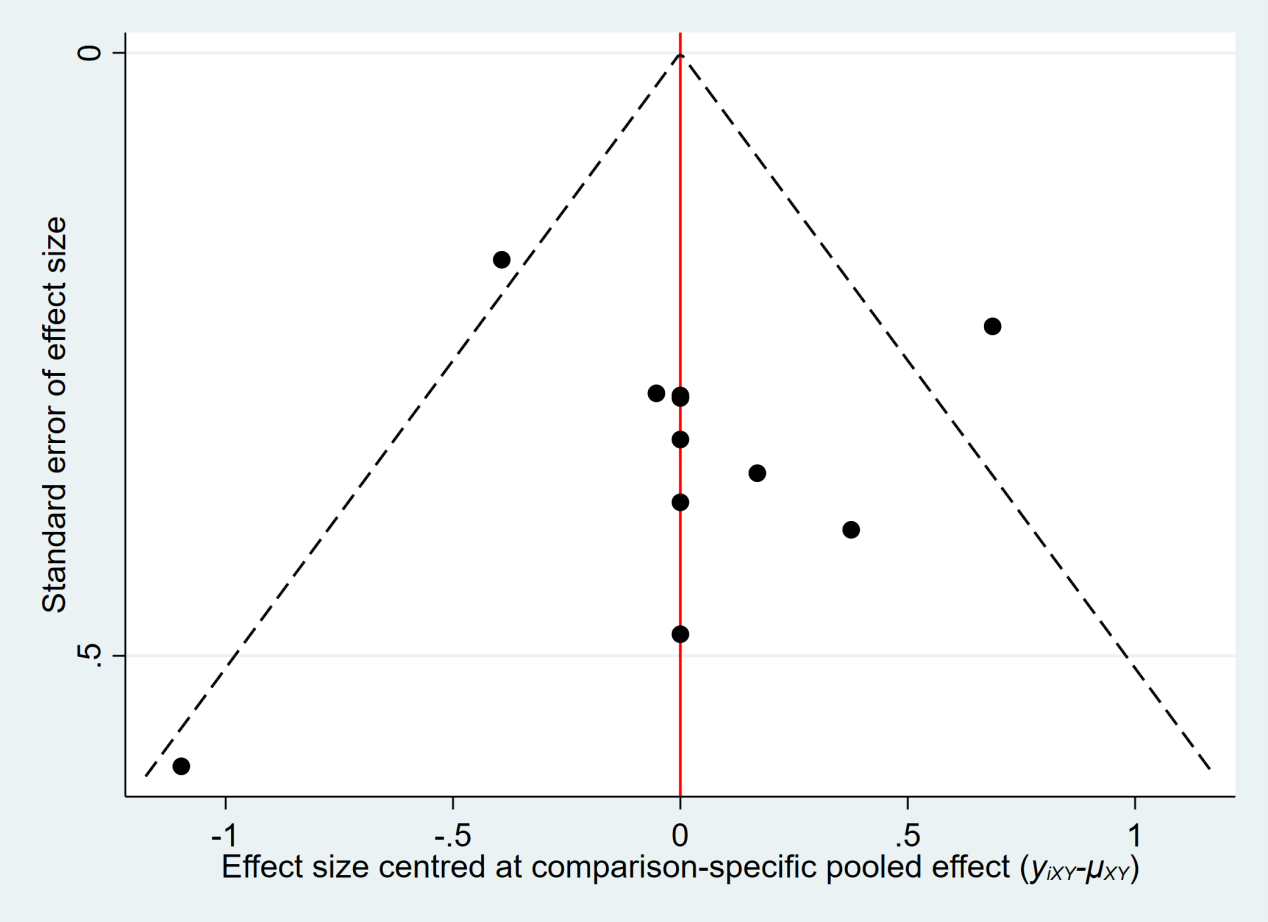


1. Serious adverse events


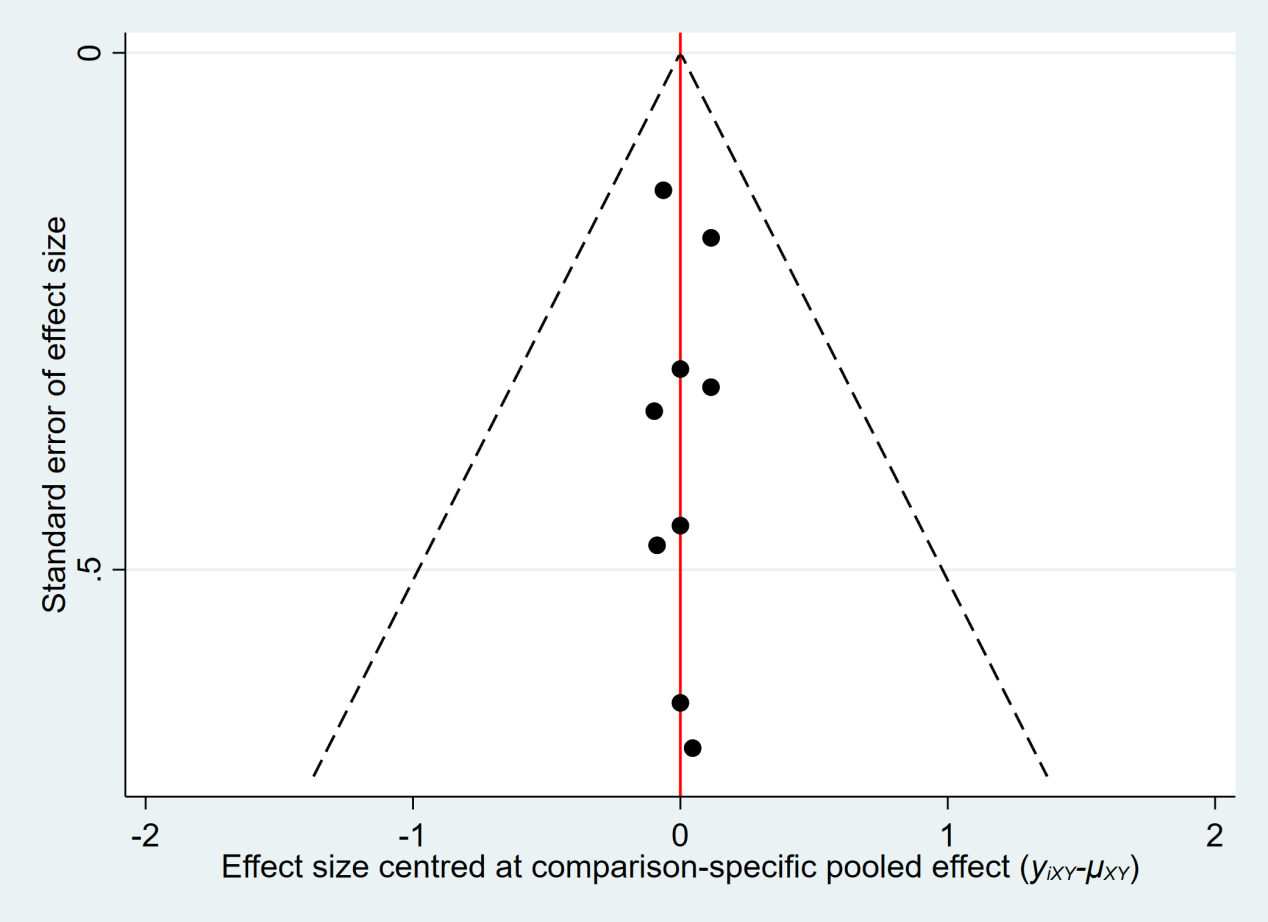

Supplement: Supplementary file 1 [file DataSheet1.docx]
